# The position of the target site for engineered nucleases improves the aberrant mRNA clearance in *in vivo* genome editing

**DOI:** 10.1038/s41598-020-61154-4

**Published:** 2020-03-06

**Authors:** Jae Hoon Lee, Sungsook Yu, Tae Wook Nam, Jae-il Roh, Young Jin, Jeong Pil Han, Ji-Young Cha, Yoon Ki Kim, Su-Cheong Yeom, Ki Taek Nam, Han-Woong Lee

**Affiliations:** 1 Department of Biochemistry, College of Life Science and Biotechnology, Yonsei University, Seoul, 03722 Republic of Korea; 2 Severance Biomedical Science Institute, Brain Korea 21 PLUS Project for Medical Science, Yonsei University College of Medicine, Seoul, 03722 Republic of Korea; 3 Graduate School of International Agricultural Technology, Seoul National University, Pyeongchang, 25354 Republic of Korea; 4 Department of Biochemistry, Lee Gil Ya Cancer and Diabetes Institute, GAIHST, Gachon University College of Medicine, Incheon, 21999 Republic of Korea; 5 Division of Life Sciences, Korea University, Seoul, 02841 Republic of Korea

**Keywords:** Genetic engineering, Gene expression

## Abstract

Engineered nucleases are widely used for creating frameshift or nonsense mutations in the target genes to eliminate gene functions. The resulting mRNAs carrying premature termination codons can be eliminated by nonsense-mediated mRNA decay. However, it is unclear how effective this process would be *in vivo*. Here, we found that the nonsense-mediated decay was unable to remove the mutant mRNAs in twelve out of sixteen homozygous mutant mice with frameshift mutations generated using engineered nucleases, which is far beyond what we expected. The frameshift mutant proteins translated by a single nucleotide deletion within the coding region were also detected in the *p53* mutant mice. Furthermore, we showed that targeting the exons present downstream of the exons with a start codon or distant from ATG is relatively effective for eliminating mutant mRNAs *in vivo*, whereas the exons with a start codon are targeted to express the mutant mRNAs. Of the sixteen mutant mice generated, only four mutant mice targeting the downstream exons exhibited over 80% clearance of mutant mRNAs. Since the abnormal products, either mutant RNAs or mutant proteins, expressed by the target alleles might obscure the outcome of genome editing, these findings will provide insights in the improved performance of engineered nucleases when they are applied *in vivo*.

## Introduction

Engineered nucleases such as clustered regularly interspaced short palindromic repeats/CRISPR-associated protein 9 (CRISPR/Cas9) and transcription activator-like effector nucleases (TALENs) are versatile tools for gene knockout^1,2^. These tools are commonly designed to cleave immediately downstream of a translation initiation codon (ATG) in the open reading frame (ORF) of target genes^1–5^. The double-strand breaks generated by cleaving DNA cause insertion/deletion (indel) mutations at the target site as a result of non-homologous end joining (NHEJ)-mediated DNA repair^5,6^. These mutations introduce premature termination codons (PTCs) in the ORF of target genes^7,8^, leading to mutant mRNA degradation by the nonsense-mediated mRNA decay (NMD)^9^. Therefore, the tools have been used to create and edit various model organisms, which provide evidence for the clinical use of the engineered nucleases^3,4^. Currently, the engineered nucleases are being developed as a therapy for treating diseases by eliminating the causative genes with harmful mutations^10^. However, more research is required to understand their effects on the treatment of the diseases.

NMD, an mRNA surveillance mechanism, largely eliminates the aberrant mRNAs containing PTCs to prevent the expression of truncated and potentially harmful mutant proteins^9^. Usually, the exon junction complexes (EJCs) bound to 20–24 bp upstream of the exon-exon junctions of mRNAs are removed by the ribosomes translating the mRNAs^9,11^. However, EJCs remain bound to the PTC-harboring mRNAs since the ribosomes translating the mRNAs stop when they reach the PTC, leading to the decay of the mRNAs through communication between the mRNA-binding EJCs and the ribosomes^9,11^. Therefore, it is widely accepted that mRNAs with the PTC in the last exon or single exon can be excluded from the decay since they do not have an EJC as a translational termination codon that is typically located in the last exon of a gene^9^. In addition, the mRNAs with PTCs located in close proximity to the AUG start codon are expected to bypass NMD owing to the AUG-proximity effect, which is probably a result of translational reinitiation initiated by the post-termination ribosomes downstream of the AUG codon^9,12^. However, it remains unclear how much mutant mRNAs generated from the alleles with frameshift mutations are eliminated *in vivo* and whether the mutant mRNAs can be translated.

This study reveals that the PTC-harboring mRNAs are frequently expressed in the mutant mice generated using the engineered nucleases such as CRISPR/Cas9 and TALENs. Specifically, the mutant p53 protein generated from the targeted allele was observed in our p53 mutant mice. Furthermore, the amount of detectable mutant mRNAs in various mutant mice was influenced by the position of mutation in the ORFs. Collectively, our findings will provide insights in the selection of a therapeutic target site using the engineered nucleases, thereby improving the current gene therapies based on PTC-mediated gene disruption using genome editors, such as Zinc-finger nucleases, TALENs, CRISPR/Cas9, and CRISPR based-editors.

## Results

### Mutant mRNAs are frequently expressed in mutant mice created by the engineered nucleases

**Figure 1:**
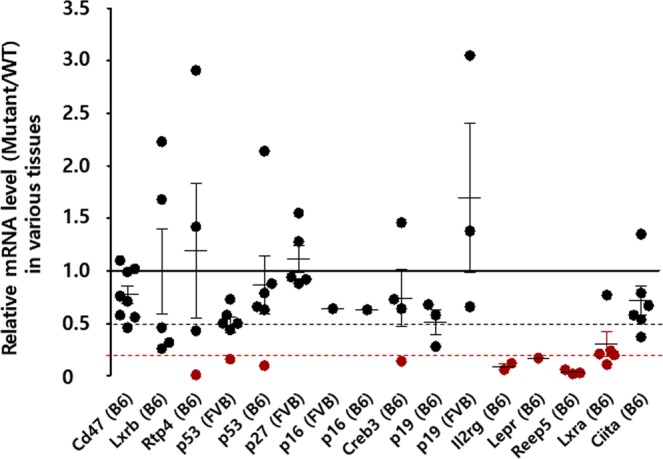
The mutant/WT ratios of the mRNA expression levels for each target gene in sixteen mutant mouse strains carrying frameshift mutations. Gene expression in different tissues of each mouse was determined using quantitative PCR (qPCR). Each closed black circles represents the target gene expression in mutant mice relative to that in WT mice in the tissues that were tested. The closed red circles indicate that the expression of target gene in mutant mice is less than 20% relative to that in WT mice. The X-axis is ordered from the shortest to the longest distance from ATG of each target gene to their indel. The middle horizontal lines in each gene of the whisker plot represent the median gene expression ratios. The dotted black line indicates 50% mutant/WT ratio, and the dotted red line indicates 20% mutant/WT ratio.

To generate sixteen knockout mice for thirteen genes by inducing indel-mediated frameshift mutations, we designed gRNAs and TALENs targeting the downstream of start codon for each gene (Supplementary Table 1). The synthesized engineered nuclease mRNAs and/or gRNAs targeting each gene were directly microinjected into the murine one-cell embryos. The pups were selected based on sequencing analysis for the target region and PTC prediction for the mutant sequences (Supplementary Tables 2 and 3). The selected mutations introduced the PTCs within the ORFs of each target gene. The mice with such mutations are typically considered as knockout mice for the specific gene due to the loss of functional target proteins. However, we hypothesized that PTC-containing mRNAs would not be completely eliminated by the NMD pathway in the mice, and therefore, evaluated the mRNA expression of each target gene in various tissues of homozygous mutant mice obtained by heterozygous intercrosses. We found that the mRNA expression of the target genes was not completely eliminated in various tissues of most mutant mice, which was more than what we expected. The ratios (KO/WT) of mutant mRNA in mutant mice to wild-type (WT) mRNA expressed in each detectable tissues of WT mice were high (>0.5) in twelve different strains for nine genes (*Cd47, Lxrb, Rtp4, p53, p27, p16, Creb3, p19*, and *Ciita*) (Fig. 1 and Supplementary Fig. 1). The NMD efficiency is diverse across different tissues. In some cases, the levels of mutant mRNA expression are more increased in mutant mice than in WT mice. In contrast to the other twelve mutant mice, the four mutant mice for *Il2rg, Lepr, Reep5*, and *Lxra* showed dramatically reduced mRNA expressions (Fig. 1 and Supplementary Fig. 1). These results suggest that even though the efficiency of NMD is variable, it can be improved by the application of specific targeting rules in the engineered nuclease-mediated indel mutations resulting in loss of gene function.

### Mutant p53 mRNAs are frequently expressed in the *p53* mutant mice created by the engineered nucleases

**Figure 2:**
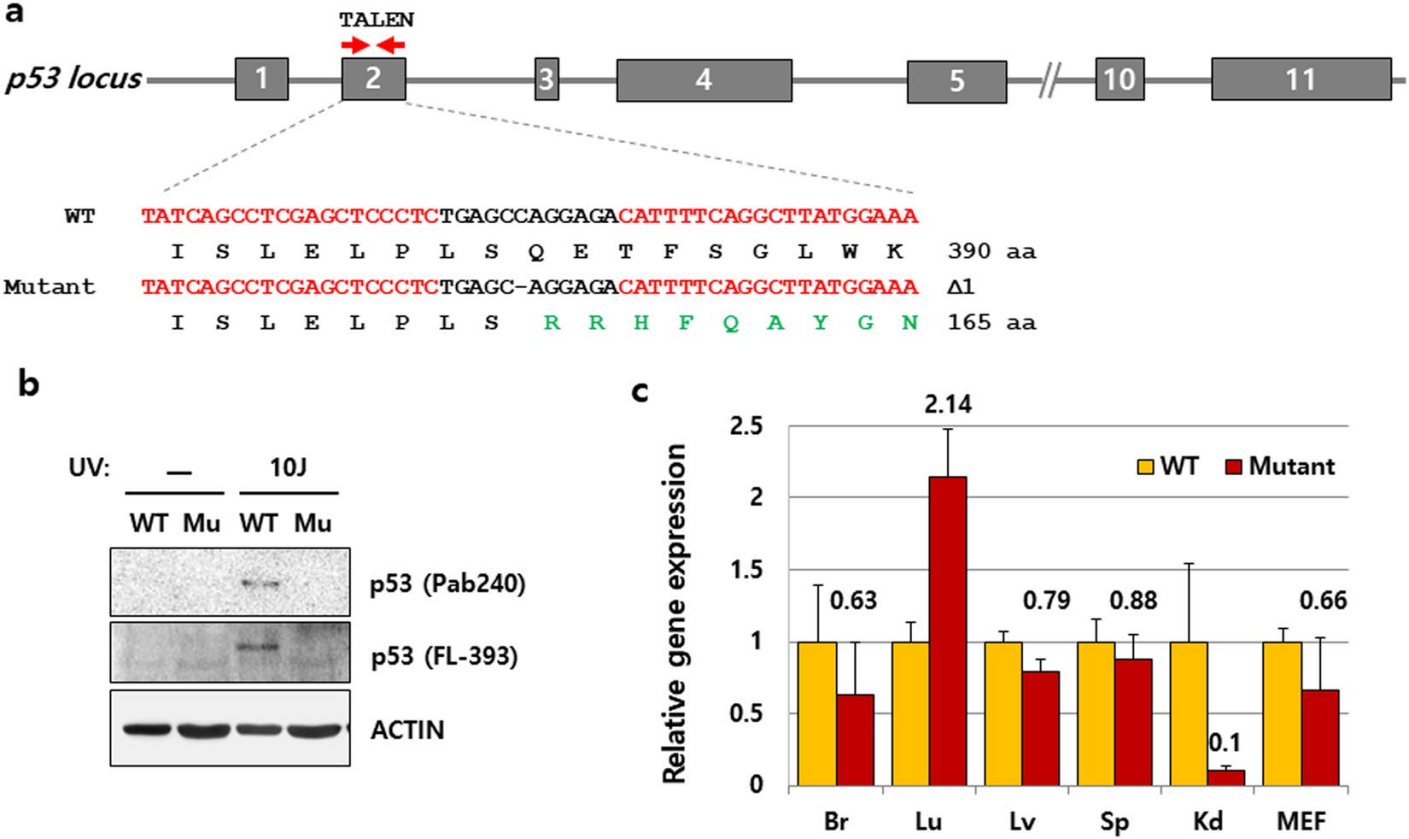
p53 mRNA expression in the *p53* mutant mice created by engineered nucleases. **(a)** Targeting strategy using a pair of TALENs on the *p53* gene. Targeting loci of two different TALENs are indicated with red arrows on exon 2 of the *p53* gene with their sequences also shown in red, determined by Sanger sequencing of PCR products from WT and *p53* mutant mice. The dash (-) denotes a deleted nucleotide; Δ1 denotes the number of deleted nucleotide(s), aa represents amino acid. **(b**,** c)** Expression of protein or mRNA encoding *p53* in mutant (Mu) MEFs induced upon exposure to 10 J/m^2^ UV treatment, determined by Western blot analysis and qPCR, respectively. *Actin* was used as an internal control. The qPCR experiments were performed in triplicates, and error bars indicate means ± standard deviation (SD). Full-length blots are depicted in Supplementary Fig. 6.

**Figure 3:**
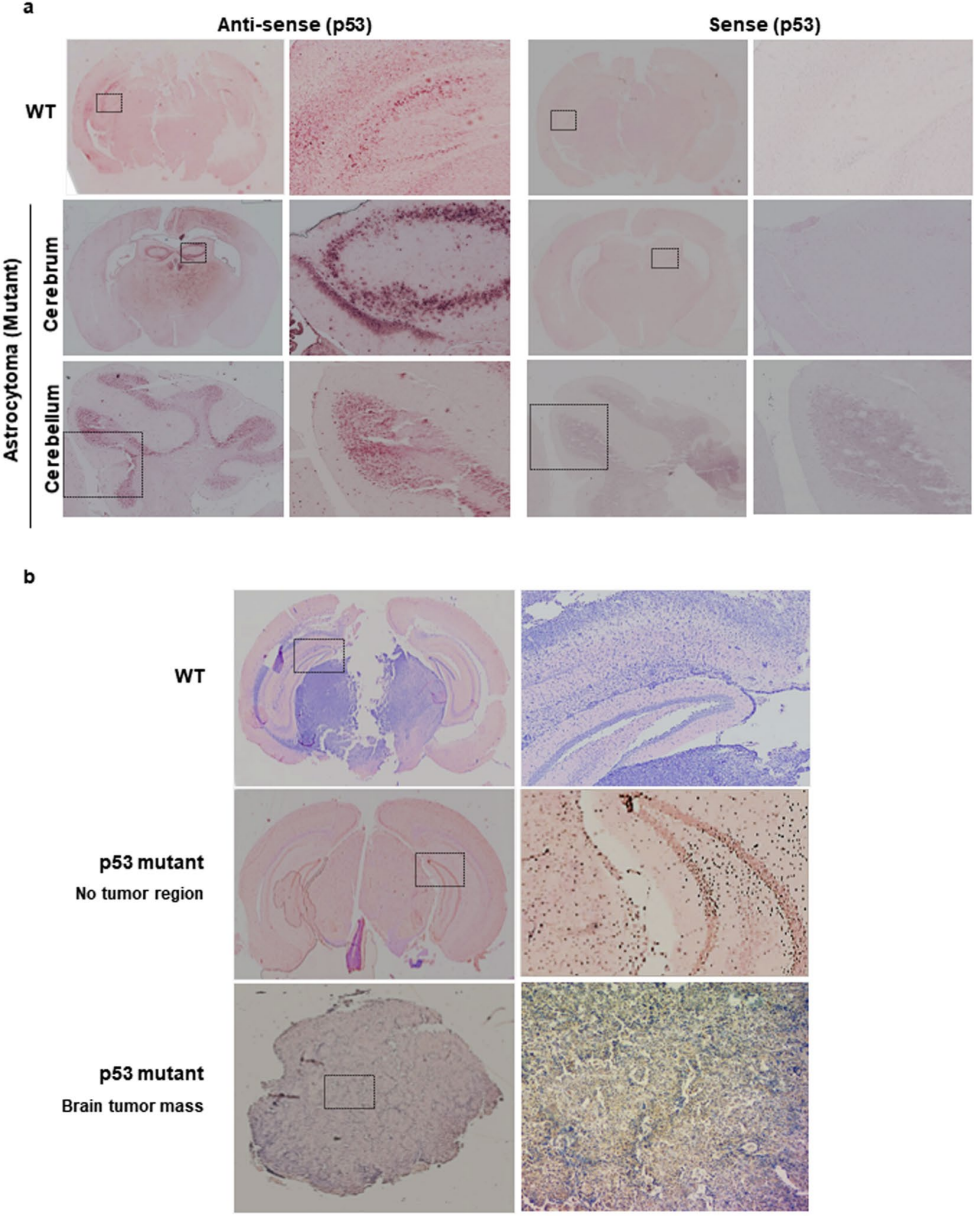
Expression of mutant p53 mRNA and protein in the brain of WT and *p53* mutant mice. (**a**) p53 mRNA in astrocytoma *in situ* in *p53* mutant mouse. DIG-labelled cRNA of p53 displayed the p53 mRNA expression in both WT and *p53* mutant mice bearing astrocytoma in the brain. **(b)** Mutant p53 protein in the brain of *p53* mutant mouse. Mutant p53 protein was detected in the brain and brain tumor of *p53* mutant mice, whereas the expression of mutant p53 was not confirmed in the brain of WT mice by immunohistochemistry using antibody against the mutant p53 protein. The enlarged parts of the dotted rectangle appear on the right panel.

Among the mutant mice that were created, *p53* mutant mice (B6J) with a single nucleotide deletion in the second exon of *p53* gene had the longest distance from the start codon to the PTC (Fig. 2a and Supplementary Table 3). Hence, these mice were selected for further experiments as detection of the resulting mutant protein was comparatively easier. The tumor suppressor protein p53 is critical for maintaining the genomic stability in multicellular organisms; its expression is therefore induced in response to DNA damage. As observed in Western blotting analysis, the induction of p53 protein expression by exposure to 10 J/m^2^ UVC was not detected in the embryonic fibroblasts of *p53* mutant mouse (mutant MEFs) (Fig. 2b). This result indicates that *p53* mutant mice were successfully generated by simple genome editing. However, as observed in RNA expression analysis, these mice expressed p53 mRNA frequently in most of the tissues that we tested (Fig. 2c). In addition, their expression levels were similar to their WT littermate, except in the lungs and the kidneys. In consistence with a previous report^13^, the efficiency of NMD varied between different tissues as the mRNA level was decreased dramatically in the kidneys, while it was increased in the lungs (Fig. 2c). Since the mutant gene expression is often avoided by *in vivo* mechanisms such as alternative splicing^14–16^, we questioned if the expression of mutant *p53* can be bypassed by alternative splicing such as nonsense-associated exon skipping. Therefore, we cloned and sequenced the cDNA from a full-length p53 mRNA which was not degraded but expressed in the *p53* mutant mice and confirmed that the RNA contained a single nucleotide excision (c.54_54del1) as expected from genomic DNA sequencing (Supplementary Fig. 2). No product of alternative splicing generated by PTC-mediated exon skipping was found under the conditions we tested. The *p53* mutant mice exhibited a similar tumor profile as ESC-based *p53* KO mice reported previously^17,18^; however, the brain tumors that were previously observed only in heterozygotes of ESC-based *p53* KO mice were observed in our homozygous *p53* mutant mice (2 out of 21 mice with tumor) (Supplementary Table 5). Furthermore, the mutant p53 mRNA was frequently detected in the brain of *p53* mutant mice with astrocytoma by *in situ* hybridization (Fig. 3a). This result indicates that the mutant p53 mRNA can be translated, though its products may be unstable, non-functional, truncated, or a gain of function mutant p53 protein.

### Mutant p53 protein is identified in *p53* homozygous mutant mice

The mutation in our *p53* mutant mice induced the frameshift from the 18^th^ amino acid of the *p53* ORF and the premature termination codon was introduced at the 165^th^ amino acid (495^th^ nucleotide) of the frameshifted *p53* ORF (Supplementary Figs. 2 and 3). Thus, the commercial antibody that we used for detection of wild-type p53 is not supposed to detect mutant p53 protein due to the alteration in the epitopes caused by frameshift mutation. To determine whether the mutant mRNA expressed in the *p53* mutant mice can indeed produce aberrant proteins, we produced an antibody for the mutant p53 protein. The mutant p53 antibody could detect the cloned mutant p53 protein overexpressed in 293 T cells (Supplementary Fig. 4). The expression of mutant p53 protein induced by UVC in homozygous mutant MEFs was also analysed to confirm it as the expected mutant protein by LC-MS/MS analyses (Supplementary Fig. 5). Furthermore, the p53 mutant protein, which was not detected in the brain of WT mice, was detected in the brain tumor mass of the *p53* mutant mice by immunohistochemistry, using the antibody against the mutant p53 protein (Fig. 3b). Although it is not yet clear whether the mutant p53 proteins can cause brain tumors, these results demonstrate that the mutant proteins can be translated from the mutant mRNAs escaping NMD in the tissues of mutant mice.

### NMD efficiency is dependent on the position of the target site in the gene

**Figure 4:**
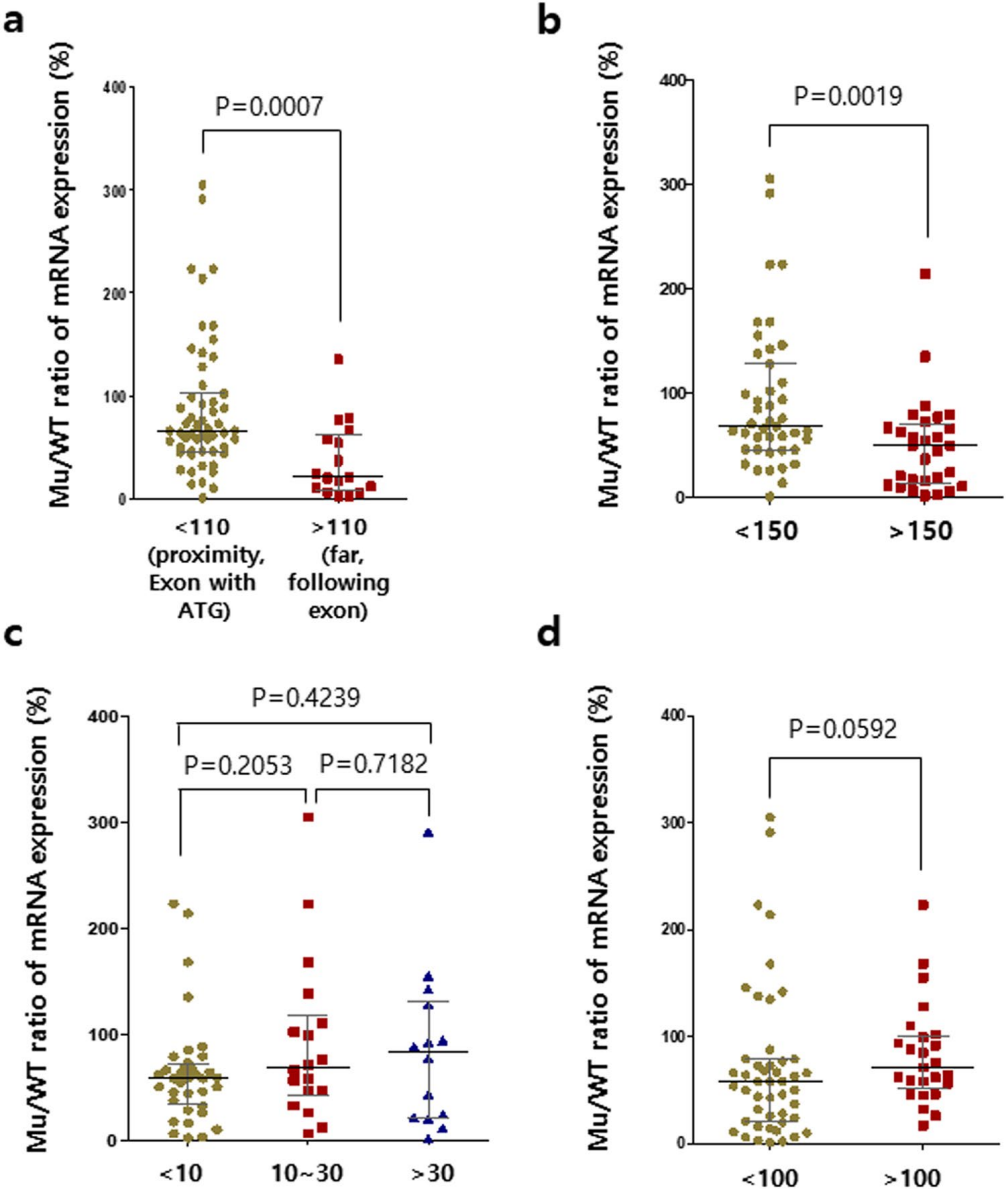
Target regions and distances from ATG codon to the PTC influence the efficiency of mutant mRNA decay (mutant mRNA levels/WT mRNA levels). The Mu/WT ratios of mRNA expression levels for target gene in each tissue are shown. **(a)** The efficiencies of mRNA decay are differentially influenced by the target regions: Proximity to ATG (<110 bp), far from ATG (>110 bp). (**b**) The influence of distance from the ATG codon to the PTC on the efficiency of mRNA decay. PTCs are located within 150 bp downstream from ATG (<150) or at distance of over 150 bp from ATG (>150). (**c**) The influence of the number of deleted nucleotides on the efficiency of mRNA decay. The Mu/WT ratios of mRNA expression levels for the target genes in each tissue are shown for each range of deletion lengths. Deletion lengths are less than 10 bp (<10 bp), from 10 to 30 bp (10~30), or over 30 bp (>30). (**d**) The distance from the PTC to the next EJC influences the efficiency of mRNA decay. PTCs are located within 100 bp upstream from the next EJC (<100) or at a distance of over 100 bp from the next EJC (>100). The central lines represent the median gene expression ratio. *p* values are from two-tail Mann-Whitney tests. Mu, Mutant.

Since the diverse genomic profiles of the sixteen mutant mice allowed us to examine the efficiency of NMD *in vivo*, we attempted to find an effective approach to improve the NMD efficacy and the mutant mRNA clearance yield induced by simple targeting using engineered nucleases. We analyzed the property of genetic mutation for each mutant mouse and compared its mRNA expression (Supplementary Table 3). There are variations in mutant mRNA clearance depending on the target position. In contrast to what is previously known, the elimination of mutant mRNAs was less effective in the mutant mice targeting exon with a start codon than in the mutant mice targeting the exons that are downstream of exons with a start codon. The ratios (KO/WT) reflecting NMD levels were less than 50% in all eleven mice targeting the exon with start codon, but 80% or more in four out of five mutant mice targeting the downstream exons (Fig. 1). In greater depth, in the eleven strains targeting the exons with start codon, the target sites were located in the regions close to the translation initiation sites (within approximately 100 bp downstream of ATG) in the common first coding exons transcribed by all known mRNA isoforms (Supplementary Table 3). In five genes, the target sites were located at a distance farther than approximately 100 bp away from the start codons, thereby avoiding the exons with start codon (Supplementary Table 3). All single gRNAs induced small deletions (indel size) within 50 bp in length, except for p27 in which 86 bp was deleted. The indel-mediated PTCs were generated within approximately 100 bp downstream from the deletion sites (distance from indels to PTC), except in *p53* and *Lxra*. As a result, the mutant mRNAs were more frequently detected in the mice where the target sites were in close proximity to ATG (<110 bp) in the genes of interest than in those where the target sites were located far away from ATG (>110 bp, Median KO/WT ratios of mRNAs: 66% vs. 21% (proximity vs. far), *p* = 0.0007, two-tailed Mann–Whitney test, Fig. 4a and Supplementary Table 3). Specifically, the abundance of mRNA in *Il2rg*, *Lepr, Reep5 and, Lxra* mutant mice was remarkably reduced by over 80% as compared to those in their WT controls of all mouse strains, except in the colon tissue of *Lxrα* mutant mice (Fig. 1 and Supplementary Fig. 1). We also found that the expression of mutant mRNAs was significantly decreased when the PTCs were located farther than 150 bp away from ATG in its mRNA sequences (50% vs. 68% median KO/WT ratio for PTCs over 150 bp vs. within 150 bp from ATG, *p* = 0.0019; Fig. 4b). The lengths of the deleted regions (indel size) and the distance from PTC to EJC did not have an effect on mutant mRNA clearance (Fig. 4c, d and Supplementary Table 3). We could not find any rule in the number of EJCs (exon-exon junctions between PTCs and the last exons) bound to PTC-harboring mRNAs. There was no difference in the genetic backgrounds of the mice (FVB/N and C57BL/6 in *p16, p19*, and *p53*; Fig. 1). Taken together, these results suggest that targeting the exons, which are downstream of the exons with start codons or distant from ATG, is effective for complete induction of NMD processes *in vivo* via engineered nucleases.

## Discussion

In this study, we aimed to determine the validity of mutant mice developed using the TALEN or CRISPR/Cas9 system by examining the NMD efficiency for PTC-containing mRNAs *in vivo*. However, these results ultimately indicated that the previous strategy to induce the PTC-mediated loss of gene function could be incomplete. The PTC-containing mRNAs might be protected from NMD in all of our mutant mice that were produced by introducing PTCs in proximity to the translation initiation site. It is possibly due to the unusual translation that allows ribosomes to reinitiate translation at the downstream AUG codons rather than at the first AUG codon^19,20^. The reinitiation of NMD-resistant transcripts could in turn lead to the expression of aberrant proteins retaining deleterious gain-of-functions, followed by the development of nonsense mutation-mediated diseases and misinterpretation of their phenotypes in the frameshifted mutant mice. Furthermore, our results as well as previous studies have shown that NMD efficiency is diversely modulated by cell types, cellular signalling, and stresses, which might obscure its ability to eliminate mRNAs (Fig. 1)^21,22^. In addition, several recent studies show that CRISPR-induced PTCs are also linked to alternative splicing and/or exon skipping to remove the exons with PTCs in the transcripts^14,15,21–23^. Accordingly, aberrant mRNAs could be maintained by either NMD-escaping or exon skipping at the exon with PTC. Thus the mutant mRNA detected in mice may mitigate the expected loss-of-function and the phenotypes of mutant mice. These results indicate that the use of the engineered nucleases to introduce PTCs in genes will pose new challenges in the field of genome editing including CRISPR based editors.

Most previous studies using engineered nucleases to produce knockout mice have targeted the regions close to a translation initiation site (ATG) or the first coding exons transcribed into all known mRNA isoforms (Supplementary Table 4)^5^. As a result, the PTCs were generated an average of 76 bp away from the deletion sites. According to our findings, targeting the regions close to the AUG codons is likely to cause the mRNAs containing PTCs to escape the NMD. Thus, the mutant RNAs may be translated to produce truncated or frameshifted proteins with deleterious gain-of-function^9,24,25^, thereby obscuring the phenotypic study of mutant mice created using the engineered nucleases. However, the expression of virtually all of the corresponding mRNAs has not been examined in many previous studies (Supplementary Table 4). One limitation of our study was that we could not detect the mutant proteins except for p53 due to the lack of antibodies against them. Further studies on deleterious gain-of-function for each mutant protein in various mutant mice are required to improve our findings. Nevertheless, the results now suggest that the measurement of aberrant mRNAs in the mutant mice created using the engineered nucleases will allow us to obtain more clear information in terms of phenotypic interpretation. Furthermore, by determining a better target position to avoid the first exons, the NMD can be induced efficiently to eliminate the mutant mRNAs expressed by the targeted alleles. Targeting the exons that are downstream of the exons with start codons or distant from ATG will improve the efficiency of the NMD process *in vivo*. In addition, we are also concerned about the possibility of nonsense-associated exon skipping (NAS). Similarly to the NMD, the NAS is bypassed when PTCs emerge at the first and last exons, but mainly appears when the exons carrying PTCs are in frame^14,15^. Although we could not confirm directly, we took separate steps from four mutant lines with reduced mRNA to prevent misinterpretation by the NAS. The primers were designed to avoid binding to the exons containing PTCs to prevent misinterpretation of the reduced signals. As a result, no exon skipping was found in these mutant mice. Taken together, inducing PTCs in the out-frame exon (except the first exon) potentially prevents aberrant protein expression by reducing exon skipping and promoting NMD.

The diverse rules and variable efficiency of NMD have also been observed in human cancers^26^. Since the efficacy of NMD is often associated with symptoms of diseases caused by mutations inducing addition of PTCs, different tumor spectrums are observed, for example, in the cancer patients with different mutations^24,27^. Therefore, the translation of mutant RNAs may affect the tumorigenic phenotypes of *p53* mutant mice by causing deleterious gain-of-function in the frameshifted mutant protein. However, there were no frameshift mutant *p53* mice models to mimic human patients. These types of models can be applied as new experimental models to study the cancer with genetic variation caused by indel mutations. Consequently, the residual mRNAs resulting from NMD bypass or exon skipping can cause unexpected problems in analyses and interpretation; for example, in the clinical trials using the CRISPR/Cas9 system to disrupt genes in patients. Along with the off-target effects of using the CRISPR/Cas9 system that have garnered great attention, incomplete RNA decay should also be one of the major concerns that needs to be accounted for when applying engineered nucleases in general gene function studies. When only one gRNA can be used in the clinic, the key step for success would lie in the efficiency of the specific gRNA that causes the loss of function of a gene. Therefore, investigators should put in more efforts into analyzing the status of NMD bypass and/or exon skipping in their trials, not only at the DNA level but also at the RNA and protein levels, to validate the lack of expression of non-natural RNAs and proteins. Thus, we believe that the findings from this study will provide a new important perspective in the field of targeted genome editing using engineered nucleases.

## Methods

### Preparation of *CRISPR/Cas9* and *TALEN* mRNAs

*Cas9* and *TALEN* mRNAs were prepared using the mMESSAGE mMACHINE T7 Ultra kit (Ambion), and sgRNAs were synthesized from PCR-driven templates using the MEGA shortscript T7 kit (Ambion) following the manufacturer’s instructions. Each mRNA was diluted in diethyl pyrocarbonate (Sigma, St. Louis, MO, USA)-treated injection buffer (0.25 mM EDTA, 10 mM Tris, pH 7.4) to the working concentrations. Plasmids encoding *Cas9* and *TALEN* were obtained from ToolGen, Inc. (Republic of Korea). The target sequences of TALENs and sequences of sgRNAs for each target gene in are displayed in Supplementary Table 1.

### Animals

All animal experiments were performed in accordance with the Korean Food and Drug Administration (KFDA) guidelines. Experimental protocols were reviewed and approved by the Institutional Animal Care and Use Committee (IACUC) of the Laboratory Animal Research Center at Yonsei University (Permit Number: 201506-322-02). Mice were maintained in the specific pathogen-free (SPF) facility of the Yonsei Laboratory Animal Research Center.

### Microinjection into mouse embryos

C57BL/6JBomTac and FVB/NTac mouse strains were used as embryo donors. The superovulation was induced by intra-peritoneal injections of 5 IU pregnant mare serum gonadotropin (Sigma) and 5 IU human chorionic gonadotropin (Sigma) at 48 hr intervals in 6–8-weeks-old female mice. The superovulated female mice were mated with stud males, and fertilized embryos were collected from the oviducts. For the generation of endonuclease-mediated mutants, microinjection of fertilized zygotes was performed as previously described^3,4^. In brief, endonucleases were injected into the cytoplasm of one-cell embryos using a piezo-driven manipulator (Prime Tech. Ltd., Japan), followed by transfer into the oviducts of ICR pseudo-pregnant foster mothers to produce live mice. For TALEN, 50 ng/μL of *TALEN* mRNA was intra-cytoplasmically injected into the mouse embryos. For CRISPR/Cas9, 20 ng/μL of *Cas9* mRNA and 100 ng/μL of sgRNA were mixed and injected into the cytoplasm of one-cell stage embryos.

### Genotyping and sequence analyses

To screen founder mice with endonuclease-mediated mutations, polyacrylamide gel electrophoresis (PAGE)-based assays were performed as previously described^28^ using genomic DNA samples from tail biopsies. In brief, the genomic region encompassing the endonuclease target site was PCR-amplified, melted, and re-annealed to form heteroduplex DNA, and loaded and analyzed by acrylamide gel electrophoresis. For sequence analysis, the PCR products from the founder mice were cloned using T-Blunt PCR Cloning Kit (SolGent Co., Ltd. Korea), and the mutations were identified by direct sequencing analysis (Cosmo Biotech Co., Ltd. Korea). To confirm the germ-line transmission of mutations, all the mutant sequences were also validated in the offspring derived from each founder. The homozygous mutant mice generated by interbreeding heterozygotes were screened by PAGE- or PCR-based genotyping methods with primer pair that distinguishes WT and mutant allele (Supplementary Table 6).

### RT-PCR and qPCR

Total RNAs from the cells and tissues of each mutant mouse were prepared with Trizol reagent (Invitrogen, Carlsbad, CA, USA), and 1 μg of total RNA was reverse-transcribed to produce cDNA using Superscript III First-Strand Synthesis System with oligo-dT primers (Invitrogen, Carlsbad, CA, USA) according to the manufacturer’s procedures. PCR was performed with Master preMix (Super Bio Co., Korea) on the MyCycler Thermal Cycler (Bio-Rad, Hercules, CA, USA). Quantitative analysis of gene expression was performed using SYBR Green SuperMix (Bio-Rad) on the CFX connect Real-Time System (Bio-Rad), with each sample measured in triplicates. The relative gene expression values were calculated using the CFX manager software (Bio-Rad) after normalization to the expression levels of *actin, Gapdh*, or *Hprt*. Means ± standard deviations were obtained from triplicate experiments. The primer pairs used in these assays are listed in the Supplementary Table 7.

### Western blotting

To analyze the p53 protein expression, cells were suspended in lysis buffer (50 mM Tris-HCl, pH 7.4, 150 mM NaCl, 20 mM EDTA, 0.2% NP-40, and protease inhibitors (GenDEPOT, TX, USA). The protein lysates were analyzed by immunoblotting with antibodies specific for p53 (sc-99 and sc-6243, Santa Cruz Biotech, Santa Cruz, CA, USA), and ACTIN (Sigma). The epitopes of polyclonal or monoclonal antibody for p53 are mapped within amino acids 1–393 (FL-393) or 14–389 (Pab240) of human origin p53 protein. The anti-mutant p53 polyclonal antibody was generated by AbFrontier (Seoul, Republic of Korea) using the peptide SAPWPSTRSHST, corresponding to the C-terminus of the frameshifted mutant p53 protein (Supplementary Fig. 4).

### *In situ* hybridiation (ISH)

Full-length *p53* sequences derived from a pEGFP-C2 p53 plasmid were used to synthesize the complementary RNA (cRNA) probes for p53. The plasmid was digested with restriction enzymes, and then labeled with Digoxigenin (DIG) by *in vitro* transcription using the RNA labeling kit (Roche Diagnostics, Indianapolis, IN, USA). Four-micrometer sections obtained from paraformaldehyde-fixed and paraffin-embedded tissue samples were deparaffinised, rehydrated, and incubated with 0.5% acetic anhydride solution to remove nonspecific binding. Hybridization was performed with DIG-sense and -antisense cRNA probes in 20× SSC solution containing 50% formamide at 42 °C overnight. The next day, the slides were washed in the SSC solution at 50 °C and incubated with an anti-DIG Fab antibody (Roche Diagnostics) conjugated to alkaline phosphatase (AP), at 4 °C overnight. To detect alkaline phosphatase, nitro blue tetrazolium/5-bromo-4-chloro-3-indolyl phosphate (NBT/BCIP, Roche Diagnostics) was added. Nuclei were counterstained with Nuclear Fast Red (Vector Laboratories, Inc., Burlingame, CA, USA).

### Immunohistochemistry (IHC)

IHC was performed as described previously^29^ with minor modification. Briefly, slides were deparaffinised and rehydrated through series of graded ethanol solutions. Antigen retrieval was performed using a pressure cooker. The endogenous peroxidase activity was blocked by incubating the slides with 3% H_2_O_2_ for 30 min followed by incubation with protein blocking solution (Dako, Glostrup, Denmark) for 1 hr at room temperature. Next, the slides were at first incubated with the mutant p53 antibody in a humid chamber at 4 °C overnight, and were then incubated with the secondary rabbit IgG (Dako) antibody for 15 minutes at room temperature, and developed with Dako Envision+ System-HRP DAB (Dako). After counterstaining with Meyer’s Hematoxylin (Sigma-Aldrich), the slides were mounted with the mounting solution (Electron Microscopy Sciences, Hatfield, PA, USA).

## Supplementary information

Supplementary information.

## Data Availability

No datasets were generated or analyzed during the current study.
