# Supplementary material for: The position of the target site for engineered nucleases improves the aberrant mRNA clearance in in vivo genome editing
Source: Sci Rep. 2020 Mar 6;10:4173. doi: 10.1038/s41598-020-61154-4 (PMC7060192; doi:10.1038/s41598-020-61154-4)
Supplement: Supplementary file 1 — Supplementary information. [file 41598_2020_61154_MOESM1_ESM.pdf]

The position of the target site for engineered nucleases improves the aberrant mRNA clearance in *in vivo* genome editing

Jae Hoon Lee[1,¶], Sungsook Yu[2,¶], Tae Wook Nam[1,¶], Jae-il Roh[1], Young Jin[1], Jeong Pil Han[3], Ji-Young Cha[4], Yoon Ki Kim[5], Su-Cheong Yeom[3], Ki Taek Nam[2,*], and Han-Woong Lee[1,*]

[¶]These authors contributed equally to this work.

[*]Corresponding authors.

[1]Department of Biochemistry, College of Life Science and Biotechnology, Yonsei University, Seoul 03722, Republic of Korea

[2]Severance Biomedical Science Institute, Brain Korea 21 PLUS Project for Medical Science, Yonsei University College of Medicine, Seoul 03722, Republic of Korea

[3]Graduate School of International Agricultural Technology, Seoul National University, Pyeongchang 25354, Republic of Korea

[4]Department of Biochemistry, Lee Gil Ya Cancer and Diabetes Institute, GAIHST, Gachon University College of Medicine, Incheon 21999, Republic of Korea

[5]Division of Life Sciences, Korea University, Seoul 02841, Republic of Korea

Supplementary Table 1. Target sequences of engineered nucleases for each target gene

Genes	Type of nucleases	Sequences with deletion mutation at the gene of interest	Direction
p16^{Ink4a}	TALEN	TGCATGACGTGCGGGCACTGNNNNNNNNNNNNGTTTCGCCCAACGCCCCGAA	+
p19^{Arf}	TALEN	TTCGTGCGATCCCGGAGACCNNNNNNNNNNNNCTGCGCTCTGGCTTTCGTGA	+
p53	TALEN	TATCAGCCTCGAGCTCCCTCNNNNNNNNNNNNCATTTTCAGGCTTATGGAAA	+
Cd47	CRISPR	GGAGATGTGGCCCTTGGCGGCGG	+
Ciita	CRISPR	ACTGGATGAAGAGACCCGGGAGG	+
Creb3	CRISPR	GGCTTCCTGCTAGAGGAAAGCGG	+
Il2rγ	CRISPR	AGGAGCACTGAGGTGTTCAGGGG	-
Lepr	CRISPR	AGAAGCCCCCTTCAAAGCCGAGG	-
Lxra	CRISPR	GCACGCTATGTCTGCCACAGCGG	+
Lxrb	CRISPR	AGTGTCCAGAGAACTTGTGGGGG	-
p27^{Kip1}	CRISPR	GCGGATGGACGCCAGACAAGCGG	+
Reep5	CRISPR	CCAGTGGCTGACGTACTGGGTGG	+
Rtp4	CRISPR	TGCAGGCTCCACTTGGCCCCGGG	-
The target sequences of TALENs or gRNAs are indicated in blue, and PAM sequence (NGG) are represented in red.			

Supplementary Table 2. Sequences of deletion regions observed in the frameshift mutant mice

Genes	Strains	Sequences with deletion mutation at the gene of interest	Deletion site (Δ)
***p16**^{Ink4a}*	**FVB/N**	WT GCGGCGGCCCAGGGCCGTGTGCATGACGTGCGGGCACTGCTGGAAGCCGGGGTTTCGCCCAACGCCCCGAACTCTTTCGGTCGT<br>A A A Q G R V H D V R A L L E A G V S P N A P N S F G R<br>Mu GCGGCGGCCCAGGGCCGTGTGCATGACGTGCGGGCACTG-------------------------CCCCGAACTCTTTCGGTCGT<br>A A A Q G R V H D V R A L ------------------------- P R T L S V V	**25**
***p16**^{Ink4a}*	**B6J**	WT GCGGCGGCCCAGGGCCGTGTGCATGACGTGCGGGCACTGCTGGAAGCCGGGGTTTCGCCCAACGCCCCGAACTCTTTCGGTCGT<br>A A A Q G R V H D V R A L L E A G V S P N A P N S F G R<br>Mu GCGGCGGCCCAGGGCCGTGTGCATGACGTGCGGGCACTGCTG-AAGCCGGGGTTTCGCCCAACGCCCCGAACTCTTTCGGTCGT<br>A A A Q G R V H D V R A L L - K P G F R P T P R T L S V V	**1**
***p19**^{Arf}*	**FVB/N**	WT GAGAGGGTTTTCTTGGTGAAGTTCGTGCGATCCCGGAGACCCAGGACAGCGAGCTGCGCTCTGGCTTTCGTGAACATGTTGTTG<br>E R V F L V K F V R S R R P R T A S C A L A F V N M L L<br>Mu GAGAGGGTTTTCTTGGTGAAGTTCGTGCGATCCCGGAGACCCAGGAC-GCGAGCTGCGCTCTGGCTTTCGTGAACATGTTGTTG<br>E R V F L V K F V R S R R P R T- R A A L W L S *	**1**
***p19**^{Arf}*	**B6J**	WT GAGAGGGTTTTCTTGGTGAAGTTCGTGCGATCCCGGAGACCCAGGACAGCGAGCTGCGCTCTGGCTTTCGTGAACATGTTGTTG<br>E R V F L V K F V R S R R P R T A S C A L A F V N M L L<br>Mu GAGAGGGTTTTCTTGGTGAAGTTCGTGCGATCCCGGAGACCCAGG----CGAGCTGCGCTCTGGCTTTCGTGAACATGTTGTTG<br>E R V F L V K F V R S R R P R ---- R A A L W L S *	**4**
p53	**FVB/N**	WT ATGGAGGAGTCACAGTCGGATATCAGCCTCGAGCTCCCTCTGAGCCAGGAGACATTTTCAGGCTTATGGAAACTACTTCCTCCA<br>M E E S Q S D I S L E L P L S Q E T F S G L W K L L P P<br>Mu ATGGAGGAGTCACAGTCGGATATCAGCCTCGAGCTCCCTCTGAG-------ACATTTTCAGGCTTATGGAAACTACTTCCTCCA<br>M E E S Q S D I S L E L P L S------- H F Q A Y G N Y F L Q	**7**
p53	**B6J**	WT ATGGAGGAGTCACAGTCGGATATCAGCCTCGAGCTCCCTCTGAGCCAGGAGACATTTTCAGGCTTATGGAAACTACTTCCTCCA<br>M E E S Q S D I S L E L P L S Q E T F S G L W K L L P P<br>Mu ATGGAGGAGTCACAGTCGGATATCAGCCTCGAGCTCCCTCTGAG-CAGGAGACATTTTCAGGCTTATGGAAACTACTTCCTCCA<br>M E E S Q S D I S L E L P L S- R R H F Q A Y G N Y F L Q	**1**

Cd47	**B6J**	WT CGGCGACCCCCCGGCGGCGCGGAGATGTGGCCCTTGGCGGCGGCGCTGTTGCTGGGCTCCTGCTGCTGCGGTTCAGCTCAACTA<br>M W P L A A A L L L G S C C C G S A Q L<br>Mu CGGCGACCCCCCGGCGGCGCGGAGATGTG----------------------CTGGGCTCCTGCTGCTGCGGTTCAGCTCAACTA<br>M C---------------------- W A P A A A V Q L N Y	**22**
Ciita	**B6J**	WT AGCAAGCTGTTGCAGGACATGGAACTGGATGAAGAGACCCGGGAGGCCTATGCCAACATTGCGGAACTGGATCAGTACGTGTTC<br>S K L L Q D M E L D E E T R E A Y A N I A E L D Q Y V F<br>Mu AGCAAGCTGTTGCAGGACATGGAACTGGATGAAGAGAC--GGGAGGCCTATGCCAACATTGCGGAACTGGATCAGTACGTGTTC<br>S K L L Q D M E L D E E T-- G G L C Q H C G T G S V R V	**2**
Creb3	**B6J**	WT GAGGAAAGCGGAGATTTGTGGGCTGCGACTGAGCCGGACGTGAAGGCTCCGCTGGACTTAGAG//GACTGGGAGGTAGAGGAT<br>E E S G D L W A A T E P D V K A P L D L E-//-D W E V E D<br>Mu GAGGAA-GCGGAGATTTGTGGGCTGCGACTGAGCCGGACGTGAAGGCTCCGCTG----------//---TGGGAGGTAGAGGAT<br>E E -A E I C G L R L S R T *	**1**
Il2rγ	**B6J**	WT GATTTGATCCTGACTTCTACAGCCCCTGAACACCTCAGTGCTCCTACTCTGCCCCTTCCAGAGGTTCAGTGCTTTGTGTTCAAC<br>D L I L T S T A P E H L S A P T L P L P E V Q C F V F N<br>Mu GATTTGATCCTGACTTCTACA-----------CCTCAGTGCTCCTACTCTGCCCCTTCCAGAGGTTCAGTGCTTTGTGTTCAAC<br>D L I L T S T ----------- P Q C S Y S A P S R G S V L C V Q	**11**
Lepr	**B6J**	WT CCTGCTGGAGCCCCAAACAATGCCTCGGCTTTGAAGGGGGCTTCTGAAGCAATTGTTGAAGCTAAATTTAATTCAAGTGGTATC<br>P A G A P N N A S A L K G A S E A I V E A K F N S S G I<br>Mu CCTGCTGGAGCCCCAAACAATGC-----CTTTGAAGGGGGCTTCTGAAGCAATTGTTGAAGCTAAATTTAATTCAAGTGGTATC<br>P A G A P N N A----- F E G G F *	**5**
Lxra	**B6J**	WT TTCCGCCGCAGTGTCATCAAGGGAGCACGCTATGTCTGCCACAGCGGTGGCCACTGCCCCATGGACACCTACATGCGGATGGGG<br>F R R S V I K G A R Y V C H S G G H C P M D T Y M R R K<br>Mu TTCCGCCGCAGTGTCATCAA---TGGACACCTACATGCGGATGGGG<br>F R R S V I N--- G H L H A D G	**41**
Lxrb	**B6J**	WT TTCGTGACCCACTATGTCTTCCCCCACAAGTTCTCTGGACACTCCCGTGCCTGGGAATGGTTCTCCTCAGCCCAGTACCTCCGC<br>M S S P T S S L D T P V P G N G S P Q P S T S A<br>Mu TTCGTGACCCACTATGTCTTCCCCCA--AGTTCTCTGGACACTCCCGTGCCTGGGAATGGTTCTCCTCAGCCCAGTACCTCCGC<br>M S S P --K F S G H S R A W E W F S S A Q Y L R	**2**

p27^{Kip1}	**FVB/N**	WT AACGGGAGCCCGAGCCTGGAGCGGATGGACGCCAGACAAGCGGAGCACCCCAAGCCTTCCGCCTGCAGAAATCTCTTCGGCCCG<br>N G S P S L E R M D A R Q A E H P K P S A C R N L F G P<br>Mu AACGGGAGCCCGAGCCTGGA-------------------------CACCCCAAGCCTTCCGCCTGCAGAAATCTCTTCGGCCCG<br>N G S P S L D------------------------- T P S L P P A E I S S A R	**25**
Reep5	**B6J**	WT AGTCCCAACAAAGATGATGACACCCAGTGGCTGACGTACTGGGTGGTATATGGTGTGTTCAGCATTGCCGAATTCTTCTCCGAT<br>S P N K D D D T Q W L T Y W V V Y G V F S I A E F F S D<br>Mu AGTCCCAACAAAGATGATGACACCCAGTGGCTGACGT-------GGTATATGGTGTGTTCAGCATTGCCGAATTCTTCTCCGAT<br>S P N K D D D T Q W L T -------W Y M V C S A L P N S S P I	**7**
Rtp4	**B6J**	WT GAGCAGACATTTCAAGAACTGATGCAGGAGGAGAAGCCCGGGGCCAAGTGGAGCCTGCATTTGGATAAGAACATTGTACCAGAT<br>E Q T F Q E L M Q E E K P G A K W S L H L D K N I V P D<br>Mu GAGCAG---CCTGCATTTGGATAAGAACATTGTACCAGAT<br>E Q --- P A F G *	**47**

The target sequences of TALENs or gRNAs are indicated in blue, and PAM sequence (NGG) are represented in red in the wild-type nucleotide sequences. The deleted nucleotides and PTCs are denoted by a red dash (-) and asterisk (*), respectively. Mutant amino acid sequences are denoted by underlined fonts. Mu, Mutant

Supplementary Table 3. Summary of the results of the engineered nucleases-mediated genome editing in mice

Gene name	Strain	Type of nucleases	Distance from ATG to indel (bp)*	Indel size (bp)	Distance in mRNA (bp)			Exon			
					from indel to PTC	from ATG to PTC	from PTC to EJC	with start codon	targeted	with PTC	last exon
Cd47	B6J	CRISPR	5	22	58	63	309	1	1	2	10
Lxrb	B6J	CRISPR	13	2	74	87	65	3	3	4	10
Rtp4	B6J	CRISPR	36	47	15	98	63	1	1	1	2
p53	FVB/N	TALEN	53	7	439	492	51	2	2	5	11
p53	B6J	TALEN	54	1	444	498	51	2	2	5	11
$p27^{Kip1}$	FVB/N	CRISPR	58	86	59	117	272	1	1	1	3
$p16^{Ink4a}$	FVB/N	TALEN	69	25	36	105	334	1	1	2	3
$p16^{Ink4a}$	B6J	TALEN	72	1	57	129	334	1	1	2	3
Creb3	B6J	CRISPR	75	1	36	112	44	1	1	2	9
$p19^{arf}$	B6J	TALEN	89	22	19	108	60	1	1	1	3
$p19^{arf}$	FVB/N	TALEN	104	1	25	129	60	1	1	1	3
Il2rγ	B6J	CRISPR	135	11	81	216	42	1	2	2	8
Lepr	B6J	CRISPR	170	5	19	189	176	2	3	3	19
Reep5	B6J	CRISPR	265	7	77	349	2	1	3	3	5

Lxra	**B6J**	**CRISPR**	**376**	**41**	**251**	**627**	**34**	**2**	**4**	**5**	**10**
Ciita	**B6J**	**CRISPR**	**503**	**2**	**115**	**618**	**47**	**1**	**3**	**5**	**19**

*Gene lists were sorted in order of the distance from ATG to indel (bp). Refer to the schematic diagram in Figure S3 to understand each parameter.

Supplementary Table 4. Publications for the genome editing in mice using engineered nucleases

Gene name	Type of nuclease	Targeting strategy	Distance in mRNA (bp)			Exon				Method to detect expression	Accession No.	Ref
			from ATG to indel	Indel size	from indel to PTC	with ATG	Target	with PTC	last exon			
Sepw1	TALEN	first coding exon	0	2~266	0	1	1	0	6	WB	NM_009156	1
Hprt	Cas9	first coding exon	3	124	37	1	1	3	9	NT	NM_013556	2
Pate1	Cas9	first coding exon	3	+1	71	1	1	2	5	NT	NM_001199953	3
Slx2	Cas9	first coding exon	4	8	15	2	2	2	9	WB, ICC	NM_001200013	4
Cetn1	Cas9	first coding exon	17	8	38	1	1	1*	1	NT	NM_007593	5
P11	Cas9	first coding exon	21	31	48	2	2	2	3	RT, WB	NM_009112	6
Prm1	Cas9	first coding exon	25	5	114	1	1	1*	1	NT	NM_013637	5
Wdr63	Cas9	first coding exon	27	73	34	2	2	3	23	NT	NM_172864	7
Ttc36	TALEN	first coding exon	27	2	19	1	1	1	3	WB, RT	NM_138951	8
Pate3	Cas9	first coding exon	35	1	26	1	1	2	3	NT	NM_001167592	3
Dmrt1	Cas9	first coding exon	35	+1	412	1	1	2	5	NT	NM_015826	9
Fam83h	Cas9	first coding exon	39	26	42	2	2	2	5	RT, WB	NM_001168253	10
Fabp7	Cas9n	first coding exon	42	28	37	1	1	2	4	WB	NM_021272	11
Mlkl	TALEN	first coding exon	54	2	1	2	2	2	11	WB	NM_001310613	12
Psd2	Cas9	first coding exon	65	112	5	1	1	1	4	WB, IHC	NM_001289602	13
Mlkl	Cas9	first coding exon	101	1	102	2	2	2	11	NT	NM_001310613	14
Pibf1	TALEN	first coding exon	110	2~14	1	2	2	2	18	NT	NM_029320	1

Dmrt3	**Cas9**	**first coding exon**	**121**	**8**	**333**	**1**	**1**	**1**	**2**	**NT**	**NM_177360**	[9]
Fam83h	**Cas9**	**first coding exon**	**133**	**26**	**42**	**2**	**2**	**2**	**5**	**RT, WB**	**XM_006520236**	[10]
FATS	**TALEN**	**first coding exon**	**154**	**151**	**159**	**2**	**2**	**2**	**8**	**WB**	**NM_001081331**	[15]
Tyr	**Cas9**	**first coding exon**	**230**	**1~32**	**20**	**1**	**1**	**1**	**5**	**NT**	**NM_011661**	[16]
Tet2	**Cas9**	**first coding exon**	**3008**	**8**	**104**	**3**	**3**	**3**	**11**	**NT**	**NM_001040400**	[17]
Ptch1	**Cas9**	**proximity to ATG**	**34**	**4~251**	**1**	**1**	**2**	**2**	**24**	**NT**	**NM_001328514**	[18]
Ccdc63	**Cas9**	**proximity to ATG**	**50**	**+1**	**154**	**2**	**3**	**4**	**12**	**NT**	**NM_001289809**	[7]
Notch1	**Cas9**	**proximity to ATG**	**77**	**5**	**47**	**1**	**2**	**2**	**34**	**IHC**	**NM_008714**	[19]
Jag1	**ZFN**	**proximity to ATG**	**84**	**2**	**19**	**1**	**2**	**2**	**26**	**NT**	**NM_013822**	[20]
Ccdc63	**Cas9**	**proximity to ATG**	**112**	**+1**	**154**	**3**	**4**	**5**	**13**	**NT**	**NM_183307**	[7]
Pate2	**Cas9**	**proximity to ATG**	**153**	**4**	**79**	**1**	**3**	**4**	**4**	**NT**	**NM_001033421**	[3]
Prss55	**Cas9**	**proximity to ATG**	**136**	**17**	**12**	**1**	**2**	**2**	**5**	**NT**	**NM_001081063**	[21]
Nxt2	**Cas9**	**proximity to ATG**	**140**	**101**	**5**	**1**	**3**	**3**	**4**	**NT**	**NM_172782**	[21]
Lyzl1	**Cas9**	**proximity to ATG**	**187**	**50**	**68**	**2**	**3**	**3**	**5**	**NT**	**NM_026092**	[21]
Plcd1	**Cas9**	**proximity to ATG**	**180**	**2~45**	**66**	**1**	**2**	**2**	**15**	**RT, IHC**	**NM_001293648**	[22]
Notch3	**ZFN**	**proximity to ATG**	**208**	**1**	**270**	**1**	**3**	**4**	**34**	**NT**	**NM_008716**	[20]
		**32 cases**			**Ave: 76**							

RT, RT-PCR; WB, Western blotting; IHC, Immunohistochemistry; ICC, Immunocytochemistry; NT, Not tested; Ave, Average;

*PTCs are present in the last exon.

Supplementary Table 5. Tumor spectrum of homozygous p53 mutant mice

Tissue	Diagnosis	Incidence	
**Brain**	Astrocytoma	2	3/21
	Neoplastic Perivascular cuffing	1	
**Liver**	Hypertrophy	1	13/21
	Inflammation	2	
	Leukemia	2	
	Lymphocytic leukemia	3	
	Necrosis	1	
	Histiocytic sarcoma	3	
	Malignant lymphoma	1	
	Focal lymphocytic infiltration	1	
**kidney**	Interstitial Nephritis	2	6/21
	Lymphocytic leukemia	3	
	Metaplasia	1	
**spleen**	Lymphoma	1	9/21
	Lymphocytic leukemia	2	
	Histiocytic sarcoma	2	
	Lympho sarcoma	1	
	Metaplasia	1	
	Hyperplasia	1	
	Malignant lymphoma	1	

**Pancreas**	Necrosis	1	1/21
**Heart**	Leukemia	1	2/21
	Lymphocytic leukemia	1	
**Thymus**	Thymoma Malignant	1	5/21
**Lung**	Hemangio sarcoma	1	9/21
	Leukemia	1	
	Lymphocytic leukemia	3	
	Metaplasia	1	
	Histiocytic sarcoma	2	
	Malignant lymphoma	1	
**Lymph node**	Leukemia	1	4/21
	Hemangio sarcoma	1	
	Lympho sarcoma	1	
	Histiocytic sarcoma	1	
	Lymphoma	1	
**Small intestine**	Leukemia	1	3/21
	Lymphocytic leukemia	1	
	Histiocytic sarcoma	1	
**Colon**	Lymphocytic leukemia	1	1/21
**Ovary**	Hemangio sarcoma	1	1/21
**Bladder**	Hypertrophy	1	3/21
	Hyperplasia	1	
	Lymphocytic leukemia	1	
**Utreus**	Histiocytic sarcoma	1	1/21

**Prostate**	Histiocytic sarcoma	1	1/21
**Tumor**	Hemangio sarcoma	1	5/21
	Fibro sarcoma	2	
	Histiocytic sarcoma	1	
	Malignant thymoma	1	

Supplementary Table 6. List of primers used for PAGE- or PCR-based genotyping

Target gene	Forward primer	Reverse primer	Size of PCR product	
			WT	Mutant
Cd47	GGTCGGTCGTTTCCCTTGAA	GATCCCCGAGCCACTCAC	**147**	**125**
Ciita	GCTGGAGAAAAAGCACTGGC	CATAGGCCTCCCGGGTCT (WT)	**241**	**-**
	ATGGAACTGGATGAAGAGACGG (Mutant)	CCCTCCCTTCTAGCCCTAGTT	**-**	**157**
Creb3	GTTGTGTTGGAAGCTGAGGA	CAGAGCAGTATGGGGGAT	**788**	**649**
Il2rg	CTCTCCCAGCTAACCTCCCT	GACTTCTACAGCCCCTGA (WT)	**200**	**-**
	GTCCTCATGTCCAGTGCGAA	GGAGCACTGAGGTGTAG (Mutant)	**-**	**259**
Lepr	CTGCTGGAGCCCCAAACAATGC	TTCAACAATTGCTTCAGAAGCC	**59**	**54**
Lxra	GGCTACATATTATGAATCAG	GAAGTTTTAATCCACACTCA	**514**	**473**
Lxrb	AGCCTAGAGTAGTGTGTTCC	GAGAGATAGCGACAAAGAGA	**655**	**653**
$p16^{Iink4a}$ ***(FVB)***	GAGGAGAGCCATCTGGAG	CCTTGCCTACCTGAATCG	**158**	**133**
$p16^{Iink4a}$ ***(B6)***	GTTTAATGGGTGGCTCCGGT	CGTGCGGGCACTGCTGGAAGCCGG (WT)	**220**	**-**
	GGTGTTAGCGTGGGTAGCAG	TTGGGCGAAACCCCAGC (Mutant)	**-**	**455**
$p19^{Arf}$ ***(FVB)***	GCTTCTCACCTCGCTTGTC	AGAGCGCAGCTCGCTG (WT)	**177**	**-**
	GCTTCTCACCTCGCTTGTC	GAGCGCAGCTCGCG (Mutant)	**-**	**176**

p19**^{Arf}* ***(B6)	TCCTCTCTAGCCTCAACAAC	AGGGTTTTCTTGGTGAAGTTC	**94**	**90**
p27**^{Kip1}* ***(FVB)	GATGTCAAACGTGAGAGTGT	CACTTGCGCTGACTCGCT	**180**	**155**
p53 (FVB)	ATTTCCCTACTGGATGTCCCACC	TTTCCATAAGCCTGAAAATGTCTCCTGG (WT)	**212**	**-**
	GAGCTCCCTCTGAGACATT (Mutant)	TTCTCTCAGGCAAGGGGAGGATA	**-**	**107**
p53 (B6)	ATTTCCCTACTGGATGTCCCACC	TTTCCATAAGCCTGAAAATGTCTCCTGG (WT)	**212**	**-**
	TCGAGCTCCCTCTGAGCA (Mutant)	TTCTCTCAGGCAAGGGGAGGATA	**-**	**115**
Reep5	CAGATGTAGGAGCTGTGGCA	AATGACTCCGCTTCAGCACT	**533**	**526**
Rtp4	CTCCTTTATTTCAATCCCCACAC	GATGGTTAGGGTCGCTTGTA	**648**	**601**

Mutant, specifically binds to the site of mutation in mutant alleles but not WT alleles; WT, specifically binds to the WT alleles but not to mutant alleles. Blue fonts represent the sequences downstream of deletion site in mutant alleles. Red fonts indicate the sequences that are only in the WT alleles because they were deleted in the mutant alleles.

Supplementary Table 7. List of primers used for RT-qPCR reactions

Target gene	Forward primer	Reverse primer	bp	Reference sequence
Actin	CATTGCTGACAGGATGCAGAAGG	TGCTGGAAGGTGGACAGTGAGG	**138**	**NM_007393**
Cd47	GGCGCAAAGCACCGAAGAAATGTT	CCATGGCATCGCGCTTATCCATTT	**179**	**NM_010581**
Ciita	GATCAGTACGTGTTCCAGGATACC	AGTTCAGTGAGGTCCTAGAG	**208**	**NM_007575**
Creb3	TGTTGCACCGCAAGCTTCGTG	AAGATCCCAGAGTGGCCAGTG	**206**	**NM_013497**
Gapdh	AGGTCGGTGTGAACGGATTTG	GGGGTCGTTGATGGCAACA	**95**	**NM_008084**
Hprt	CTGGTGAAAAGGACCTCTCGAAG	CCAGTTTCACTAATGACACAAACG	**146**	**NM_013556**
Il2rg	AATTCCCCCCATCAAGAATC	GTAGTCTGGCTGCAGACTCTCA	**109**	**NM_001308535**
Lepr	ACTGTGCAGCTGAGGTATCAC	GGCTGGACTGCTCCAATTACT	**507**	**NM_001122899**
Lxra	ATGCTACGCAAGGCTCTCTCC	GCAAGTGTTTGCCCTTCGCC	**203**	**NM_013839**
Lxrb	ATCTGAGCCAGCAGCCAGCA	TGACTTTGGGCTGGTCGGAGAA	**188**	**NM_009473**
$p16^{Iink4a}$	AAAGCGAACTCGAGGAGAGC	TCATCATCACCTGAATCGGGG	**169**	**NM_001040654**

***p19**^{Arf}*	CGCTCTGGCTTTCGTGAACA	TTGAGCAGAAGAGCTGCTACGT	**121**	**NM_009877**
***p27**^{Kip1}*	GCGGTGCCTTTAATTGGGTCT	GGCTTCTTGGGCGTCTGCT	**230**	**NM_009875**
p53	CCCCTGTCATCTTTTGTCCCT	TTTCCTTCCACCCGGATAAG	**326**	**NM_011640**
Reep5	GAGATGTAGGCTGGGTATCCGAA	GGTTCCTGCACGAGAAGAACTG	**181**	**NM_007874**
Rtp4	GGAAGATGTGCCCTTGCACAC	GTGGCTCTATTTCACGTTGGGG	**196**	**NM_023386**

Supplementary Figure 1

a

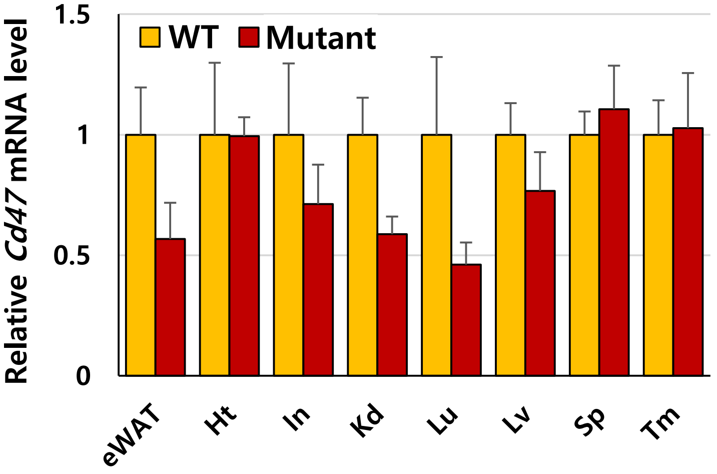

	Mu/WT
eWAT	0.56
Ht	0.99
It	0.71
Kd	0.58
Lu	0.46
Lv	0.76
Sp	1.10
Tm	1.02

b

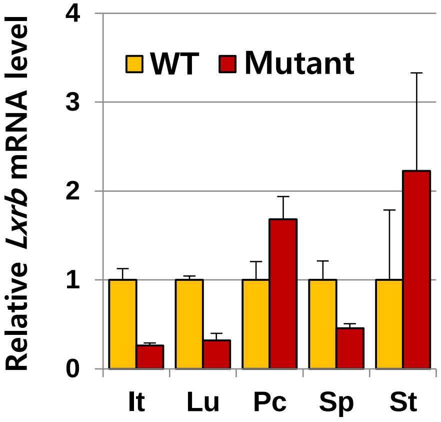

	Mu/WT
It	0.26
Lu	0.32
Pc	1.68
Sp	0.46
St	2.23

c

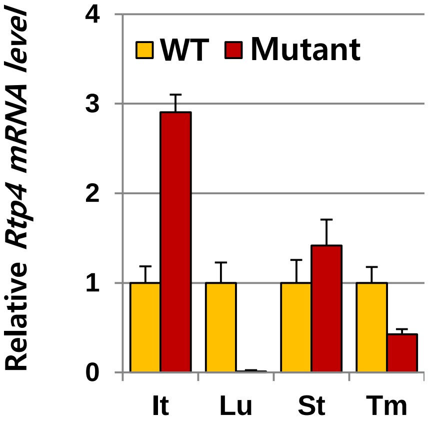

	Mu/WT
It	2.91
Lu	0.01
St	1.42
Tm	0.43

d

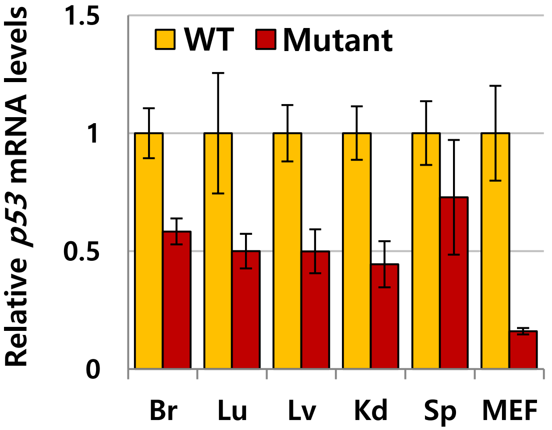

	Mu/WT
Br	0.58
Lu	0.50
Lv	0.50
Kd	0.44
Sp	0.73
MEF	0.16

Supplementary Figure 1 (continued)

e

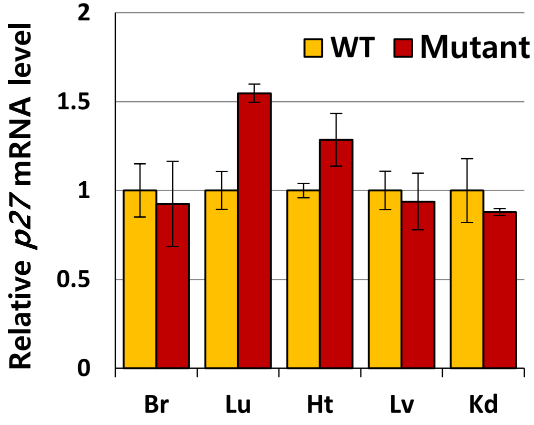

	Mu/WT
Br	0.92
Lu	1.55
Ht	1.28
Lv	0.94
Kd	0.88

f

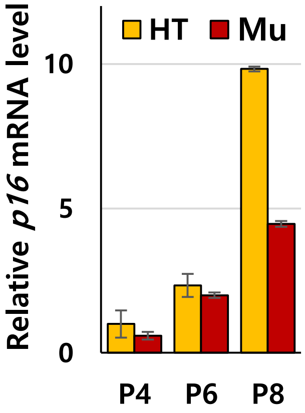

	Mu/HT
P4	0.59
P6	0.85
P8	0.45

g

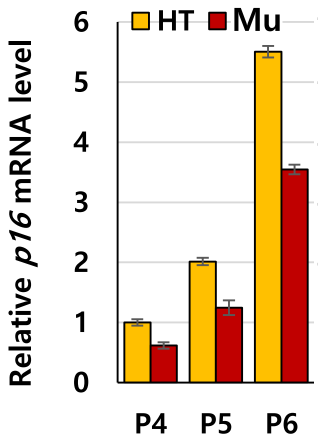

	Mu/HT
P4	0.62
P5	0.62
P6	0.64

h

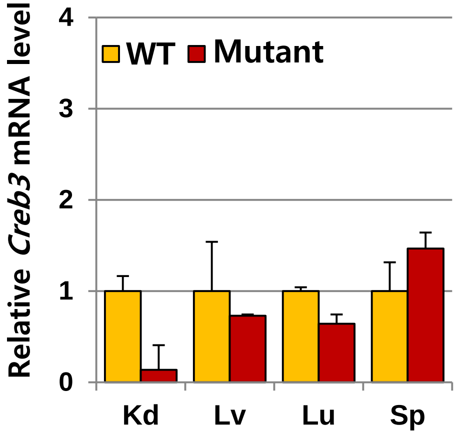

	Mu/WT
Kd	0.14
Lv	0.73
Lu	0.64
Sp	1.46

i

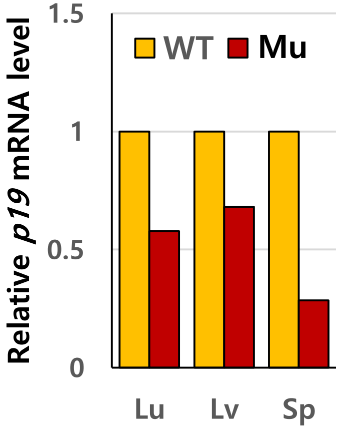

	Mu/WT
Lu	0.58
Lv	0.68
Sp	0.28

j

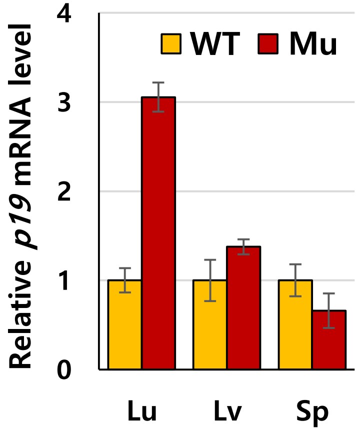

	Mu/WT
Lu	3.05
Lv	1.38
Sp	0.66

Supplementary Figure 1 (continued)

k

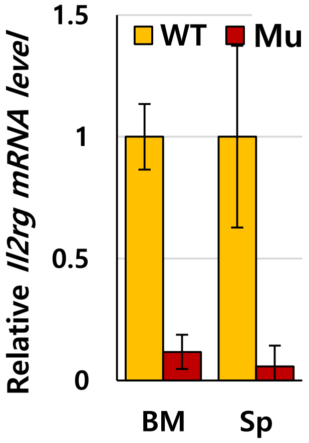

	Mu/WT
BM	0.12
Sp	0.06

l

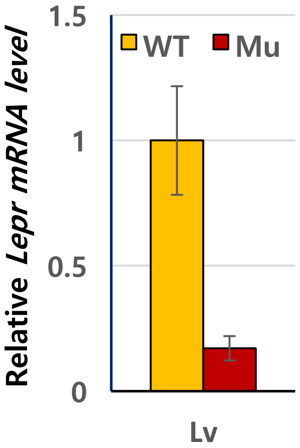

	Mu/WT
Lv	0.17

m

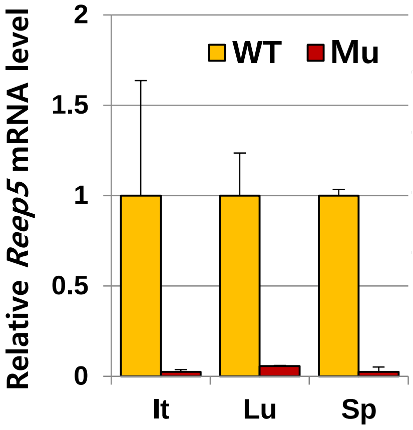

	Mu/WT
It	0.02
Lu	0.06
Sp	0.03

n

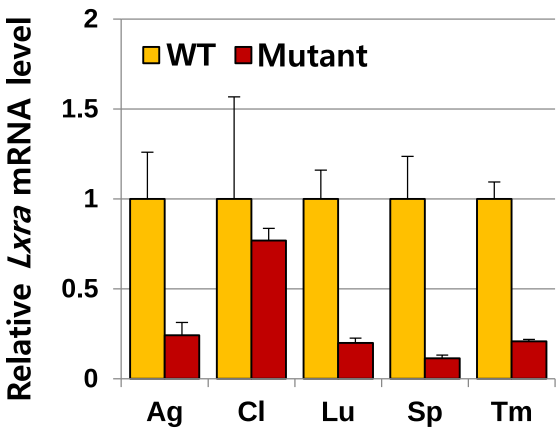

	Mu/WT
Ag	0.24
Cl	0.77
Lu	0.20
Sp	0.11
Tm	0.21

o

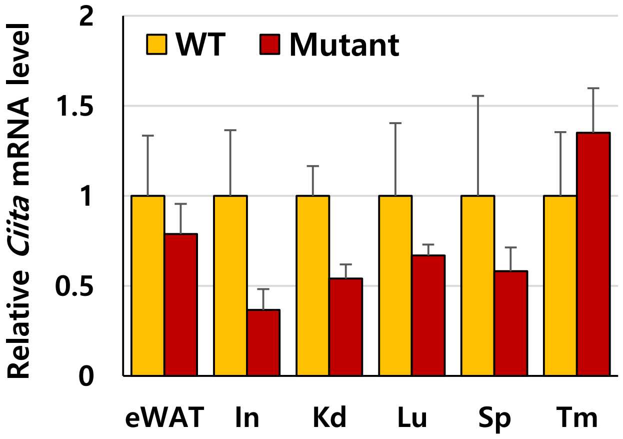

	Mu/WT
eWAT	0.79
It	0.37
Kd	0.54
Lu	0.67
Sp	0.58
Tm	1.35

Supplementary Figure 2

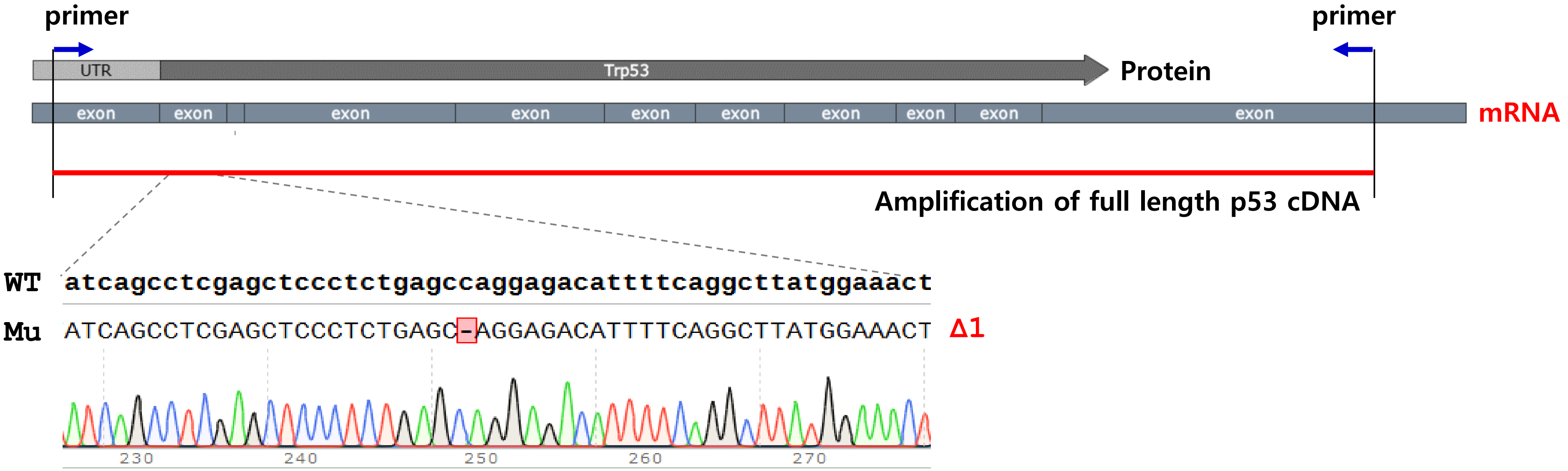

> Mutant p53 cDNA sequence (c.54_54del1)

CATCCTGGCTGTAGGTAGCGACTACAGTTAGGGGGCACCTAGCATTCAGGCCCTCATCCTCCTCCTTCCCAGCAGGGTGTCACGCTTCTCC
GAAGACTGGATGACTGCCATGGAGGAGTCACAGTCGGATATCAGCCTCGAGCTCCCTCTGAGC-AGGAGACATTTTCAGGCTTATGGAAAC
TGCTTCCTCCAGAAGATATCCTGCCATCACCTCACTGCATGGACGATCTGTTGCTGCCCCAGGATGTTGAGGAGTTTTTTGAAGGCCCAAG
TGAAGCCCTCCGAGTGTCAGGAGCTCCTGCAGCACAGGACCCTGTCACCGAGACCCCTGGGCCAGTGGCCCCTGCCCCAGCCACTCCATGG
CCCCTGTCATCTTTTGTCCCTTCTCAAAAAACTTACCAGGGCAACTATGGCTTCCACCTGGGCTTCCTGCAGTCTGGGGCAGCCAAGTCTG
TTATGTGCACGTACTCTCCTCCCCTCAATAAGCTATTCTGCCAGCTGGCGAAGACGTGCCCTGTGCAGTTGTGGGTCAGCGCCACACCTCC
AGCTGGGAGCCGTGTCCGCGCCATGGCCATCTACAAGAAGTCACAGCACATGACGGAGGTCGTGAGACGCTGCCCCCACCATGAGCGCTGC
TCCGATGGTGATGGCCTGGCTCCTCCCCAGCATCTTATCCGGGTGGAAGGAAATTTGTATCCCGAGTATCTGGAAGACAGGCAGACTTTTC
GCCACAGCGTGGTGGTACCTTATGAGCCACCCGAGGCCGGCTCTGAGTATACCACCATCCACTACAAGTACATGTGTAATAGCTCCTGCAT
GGGGGGCATGAACCGCCGACCTATCCTTACCATCATCACACTGGAAGACTCCAGTGGGAACCTTCTGGGACGGGACAGCTTTGAGGTTCGT
GTTTGTGCCTGCCCTGGGAGAGACCGCCGTACAGAAGAAGAAAATTTCCGCAAAAAGGAAGTCCTTTGCCCTGAACTGCCCCCAGGGAGCG
CAAAGAGAGCGCTGCCCACCTGCACAAGCGCCTCTCCCCCGCAAAAGAAAAAACCACTTGATGGAGAGTATTTCACCCTCAAGATCCGCGG
GCGTAAACGCTTCGAGATGTTCCGGGAGCTGAATGAGGCCTTAGAGTTAAAGGATGCCCATGCTACAGAGGAGTCTGGAGACAGCAGGGCT
CACTCCAGCTACCTGAAGACCAAGAAGGGCCAGTCTACTTCCCGCCATAAAAAAACAATGGTCAAGAAAGTGGGGCCTGACTCAGACTGAC
TGCCTCTGCATCCCGTCCCCATCACCAGCCTCCCCCTCTCCTTGCTGTCTTATGACTTCAGGGCTGAGACACAATCCTCCCGGTCCCTTCT
GCTGCCTTTTTACCTTGTAGCTAGGGCTCAGCCCCCTCTCTGAGTAGTGGTTCCTGGCCCAAGTTGGGGAATAGGTTGATAGTTGTCAGG
TCTCTGCTGGCCCAGCGAAATTCTATCCAGCCAGTTGTTGGACCCTGGCACCTACAATGAAATCTCACCCTACCCCACACCCTGTAAGATT
CTATCTTGGGCCCTCATAGGGT

Supplementary Figure 3

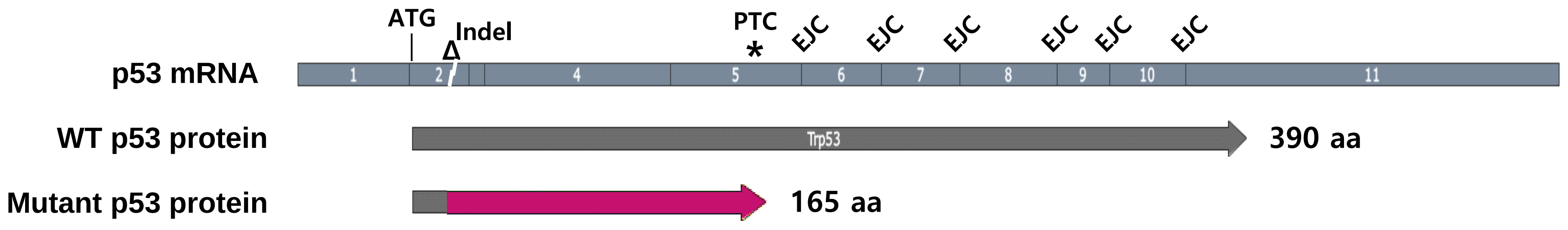


```
WT     1 MTAMEESQSD ISLELPLSQE TFSGLWKLLP PEDILPSPHC MDDLLLPQDV EEFFEGPSEA  60
Mu     1 MTAMEESQSD ISLELPLSRR HFQAYGNYFL QKISCHHLTA WTICCCPRML RSFLKAQVKP  60

WT    61 LRVSGAPAAQ DPVTETPGPV APAPATPWPL SSFVPSQKTY QGNYGFHLGF LQSGTAKSVM 120
Mu    61 SECQELLQHR TLSPRPLGQW PLPQPLHGPC HLLSLLKKLT RATMASTWAS CSLGQPSLLC 120

WT   121 CTYSPPLNKL FCQLAKTCPV QLWVSATPPA GSRVRAMAIY KKSQHMTEVV RRCPHHERCS 180
Mu   121 ARTLLPSISY SASWRRRALC SCGSAPHLQL GAVSAPWPST RSHST*                180

WT   181 DGDGLAPPQH LIRVEGNLYP EYLEDRQTFR HSVVVPYEPP EAGSEYTTIH YKYMCNSSCM 240

WT   241 GGMNRRPILT IITLEDSSGN LLGRDSFEVR VCACPGRDRR TEEENFRKKE VLCPELPPGS 300

WT   301 AKRALPTCTS ASPPQKKKPL DGEYFTLKIR GRKRFEMFRE LNEALELKDA HATEESGDSR 360

WT   361 AHSSYLKTKK GQSTSRHKKT MVKKVGPDSD*
```

Supplementary Figure 4

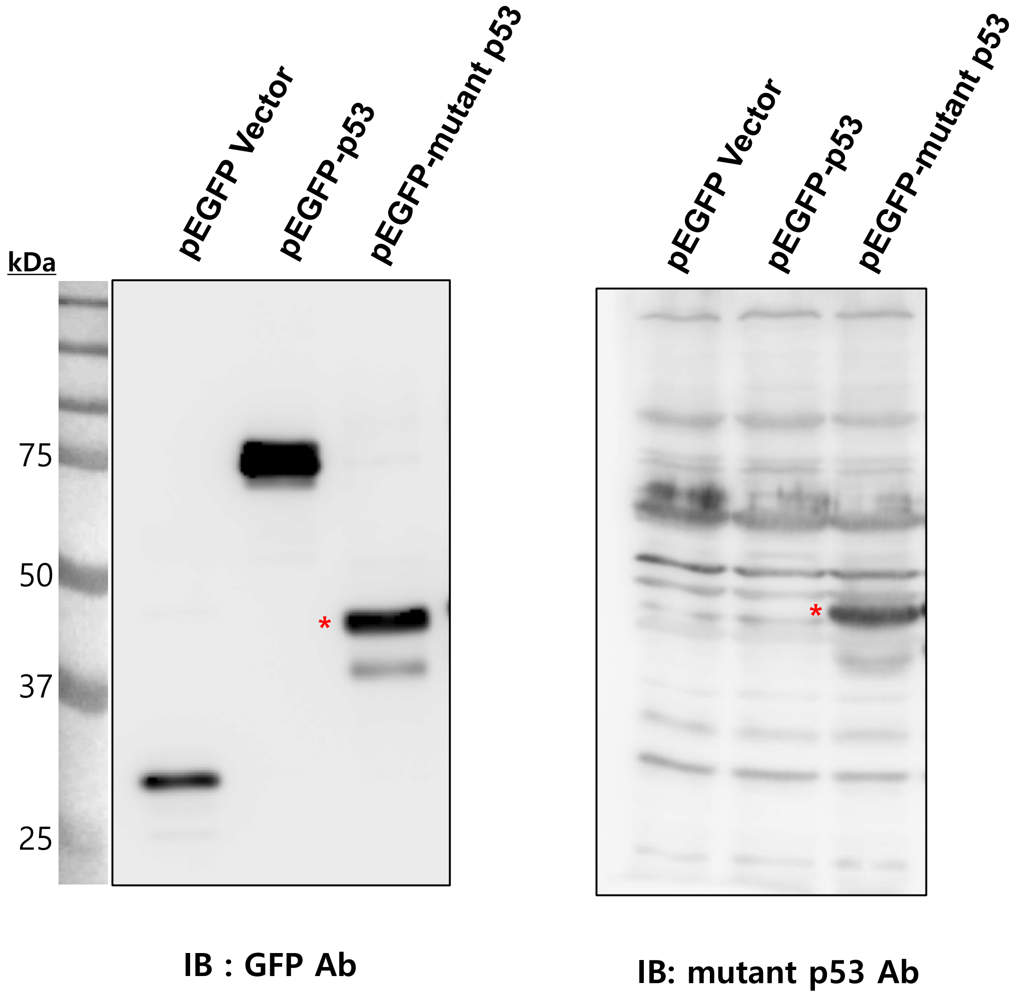

Supplementary Figure 5

Mascot Score Histogram

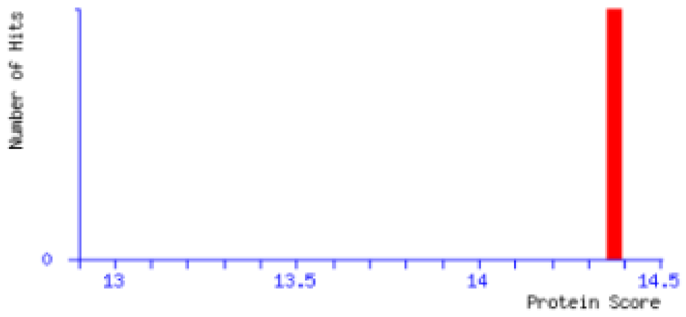

Protein sequence coverage: 27%

Matched peptides shown in ***bold red***.

```
  1 MTAMEESQSD ISLELPLSRR HFQAYGNYFL QKISCHHLTA WTICCCPRML
 51 RSFLKAQVKP SECQELLQHR TLSPRPLGQW PLPQPLHGPC HLLSLLKKLT
101 RATMASTWAS CSLGQPSLLC ARTLLPSISY SASWRRRALC SCGSAPHLQL
151 GAVSAPWPST RSHST
```

	Query	Observed	Mr(expt)	Mr(calc)	Delta	Miss	Score	Expect	Rank	Unique	Peptide
☑	550	493.6483	1477.9230	1479.7722	-1.8491	0	9	0.12	1	U	R.TLLPSISYSASWR.R
☑	589	758.8500	1515.6855	1517.6827	-1.9972	0	1	0.76	1	U	R.HFQAYGNYFLQK.I
☑	592	758.8527	1515.6908	1517.6827	-1.9919	0	(1)	0.82	1	U	R.HFQAYGNYFLQK.I
☑	1372	776.2241	2325.6504	2325.0828	0.5676	1	(12)	0.069	1	U	-.MTAMEESQSDISLELPLSRR.H + 2 Oxidation (M)
☑	1373	776.2247	2325.6522	2325.0828	0.5694	1	(5)	0.34	1	U	-.MTAMEESQSDISLELPLSRR.H + 2 Oxidation (M)
☑	1374	776.2260	2325.6562	2325.0828	0.5734	1	(3)	0.49	1	U	-.MTAMEESQSDISLELPLSRR.H + 2 Oxidation (M)
☑	1375	776.2305	2325.6696	2325.0828	0.5868	1	(8)	0.17	1	U	-.MTAMEESQSDISLELPLSRR.H + 2 Oxidation (M)
☑	1376	776.2354	2325.6844	2325.0828	0.6016	1	14	0.037	1	U	-.MTAMEESQSDISLELPLSRR.H + 2 Oxidation (M)

Supplementary Figure 6

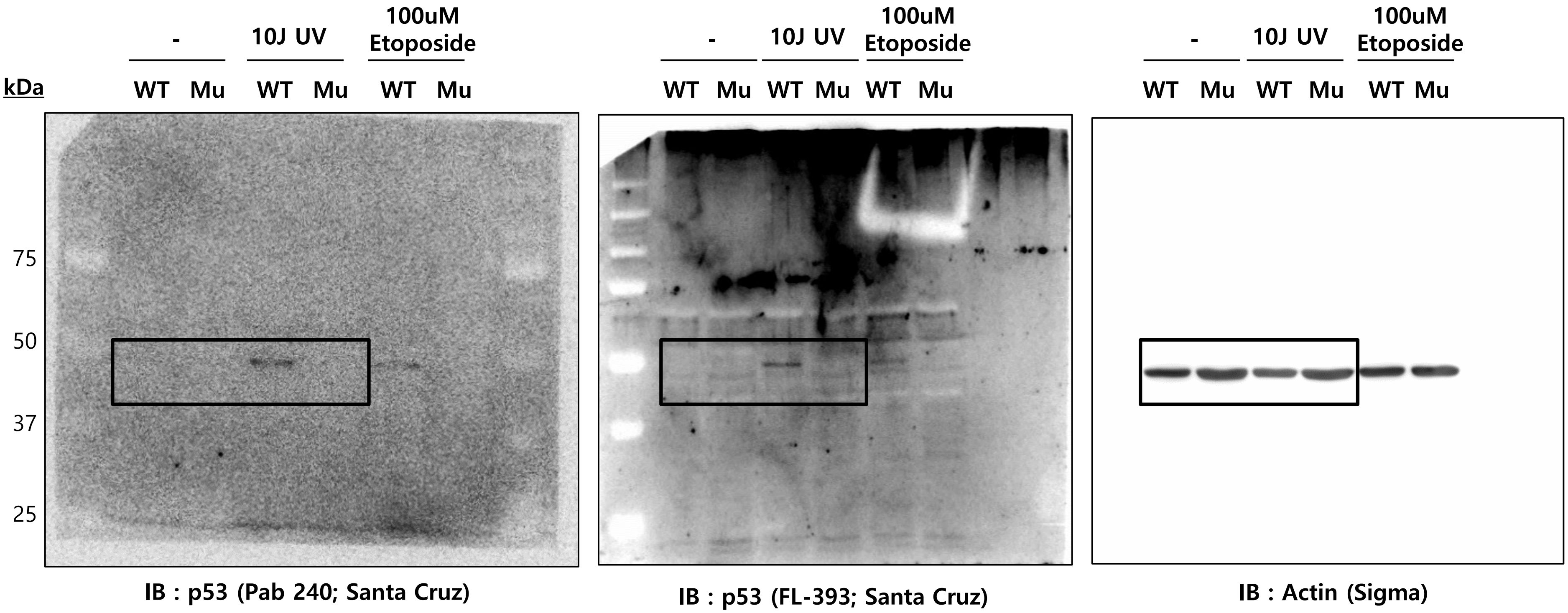

Supplementary Figure Legends

Supplementary Figure 1. Abnormal mRNAs detected in mutant mice generated by engineered nuclease. The mRNA expression levels of each gene of interest were determined by RT-qPCR according to each tissue samples. The genes are listed as follows: *Cd47* (a), *Lxrb* (b), *Rtp4* (c), *p53* (d) in FVB/N mice; $p27^{Kip1}$ (e), $p16^{Ink4a}$ (f) in C57BL/6J strain; $p16^{Ink4a}$ (g) in FVB/N strain; *Creb3* (h), $p19^{Arf}$ (i) in C57BL/6J strain; $p19^{Arf}$ (j) in FVB/N strain; *Il2rg* (k), *Lepr* (l), *Reep5* (m), *Lxra* (n), and *Ciita* (o). Numerical values of normalized ratios between wild-type (WT) and mutant (Mu) are represented next to each bar graph. The experiments were performed in triplicates, and error bars indicate means ± SD. P denotes the passage number in MEFs of $p16^{Ink4a}$ mutant mice. Ag, adrenal gland; BAT, brown adipose tissue; BM, bone marrow; Br, brain; Cl, colon; eWAT, epididymal white adipose tissue; Ht, heart; In, intestine; iWAT, inguinal white adipose tissue; Kd, kidney; Lu, lung; Lv, liver; MEF, mouse embryonic fibroblasts; Pc, Pancreas; Sp, spleen; St, Stomach; Tm, thymus.

Supplementary Figure 2. The sequence of full-length 1.5 kb *p53* cDNA cloned from the liver of *p53* mutant mice. The schematic diagram represents the p53 protein and mRNA structures. Red line denotes an amplified and cloned PCR product from the first strand of cDNA using the primers represented by blue arrows. The chromatogram shows the sequencing result for the enlarged region of *p53* sequence with deletion mutation. A dash (-) denotes a deleted nucleotide in the mutant p53 cDNA sequence (c.54_54del1). Red font and red box indicate the start codon and the premature termination codon, respectively. Mu, Mutant; WT, wild-type.

Supplementary Figure 3. The amino acid sequence of mutant *p53* which can be expressed in *p53* mutant mice. The schematic diagram represents the p53 mRNA and its mutant p53 protein sequence compared to WT p53 protein sequence. The red fonts and red box indicate the mutant p53 sequence and the epitope

region of mutant p53 antibody, respectively. Deletion mutations are noted as Δ in the mRNA structure. ATG, translational initiation codon; PTC, premature termination codon; EJC, exon-junction complex; aa, amino acid. Mu, Mutant; WT, wild-type.

Supplementary Figure 4. Western blot analysis of mutant *p53* expression in 293T cells using the mutant p53 antibody we generated. The 293T cells were transfected with the indicated plasmids (pEGFP, pEGFP-p53, and pEGFP-mutant p53). The mutant p53 antibody specifically detected a mutant p53 protein but not WT p53 protein. Left panel shows a Western blot with GFP antibody to confirm the expression of EGFP-tagged p53 and mutant p53 proteins. Asterisks indicate the mutant p53 proteins.

Supplementary Figure 5. Mascot search result for MS/MS measurements of mutant p53 protein. *p53* mutant MEFs treated with 10 J/m^2 UV were immunoprecipitated using the mutant p53 antibody, and separated by SDS-PAGE on 10% gel. The gel was stained with Coomassie Blue R250. Major protein bands were excised from the gel and identified by MS/MS. The distribution of the Mascot score for MS/MS measurements of mutant p53 protein is shown as histograms. The peptides of mutant p53 protein found in the MS/MS analysis are indicated by red fonts above the full amino acid sequence of the mutant p53 protein. The box in the figure highlights the peptide sequence of the mutant p53 protein.

Supplementary Figure 6. Full–length blots of the experiments shown in Fig. 2b. Uncropped images are boxed in the regions shown in the main text. The etoposide results were excluded from the main text.

Supplementary References

1 Sung, Y. H. *et al.* Knockout mice created by TALEN-mediated gene targeting. *Nat Biotechnol* **31**, 23-24, doi:10.1038/nbt.2477 (2013).

2 Fujii, W., Kawasaki, K., Sugiura, K. & Naito, K. Efficient generation of large-scale genome-modified mice using gRNA and CAS9 endonuclease. *Nucleic Acids Res* **41**, e187, doi:10.1093/nar/gkt772 (2013).

3 Noda, T. *et al.* Nine genes abundantly expressed in the epididymis are not essential for male fecundity in mice. *Andrology* **7**, 644-653, doi:10.1111/andr.12621 (2019).

4 Li, M. *et al.* CRISPR/Cas9 Promotes Functional Study of Testis Specific X-Linked Gene In Vivo. *PLoS One* **10**, e0143148, doi:10.1371/journal.pone.0143148 (2015).

5 Mashiko, D. *et al.* Generation of mutant mice by pronuclear injection of circular plasmid expressing Cas9 and single guided RNA. *Sci Rep* **3**, 3355, doi:10.1038/srep03355 (2013).

6 Liu, G., Wang, Y., Zheng, W., Cheng, H. & Zhou, R. P11 Loss-of-Function is Associated with Decreased Cell Proliferation and Neurobehavioral Disorders in Mice. *Int J Biol Sci* **15**, 1383-1395, doi:10.7150/ijbs.33773 (2019).

7 Young, S. A. *et al.* CRISPR/Cas9-Mediated Rapid Generation of Multiple Mouse Lines Identified Ccdc63 as Essential for Spermiogenesis. *Int J Mol Sci* **16**, 24732-24750, doi:10.3390/ijms161024732 (2015).

8 Liu, Y. *et al.* A modified TALEN-based strategy for rapidly and efficiently generating knockout mice for kidney development studies. *PLoS One* **9**, e84893, doi:10.1371/journal.pone.0084893 (2014).

9 Inui, M., Tamano, M., Kato, T. & Takada, S. CRISPR/Cas9-mediated simultaneous knockout of Dmrt1 and Dmrt3 does not recapitulate the 46,XY gonadal dysgenesis observed in 9p24.3 deletion patients. *Biochem Biophys Rep* **9**, 238-244, doi:10.1016/j.bbrep.2017.01.001 (2017).

10 Nasseri, S. *et al.* Generation of Fam83h knockout mice by CRISPR/Cas9-mediated gene engineering. *J Cell Biochem*, doi:10.1002/jcb.28381 (2019).

11 Shimamoto-Mitsuyama, C. *et al.* Evaluation of the role of fatty acid-binding protein 7 in controlling schizophrenia-relevant phenotypes using newly established knockout mice. *Schizophr Res*, doi:10.1016/j.schres.2019.02.002 (2019).

12 Wu, J. *et al.* Mlkl knockout mice demonstrate the indispensable role of Mlkl in necroptosis. *Cell Res* **23**, 994-1006, doi:10.1038/cr.2013.91 (2013).

13 Saegusa, S. *et al.* Mice lacking EFA6C/Psd2, a guanine nucleotide exchange factor for Arf6, exhibit lower Purkinje cell synaptic density but normal cerebellar motor functions. *PLoS One* **14**, e0216960, doi:10.1371/journal.pone.0216960 (2019).

14 Dannappel, M. *et al.* RIPK1 maintains epithelial homeostasis by inhibiting apoptosis and necroptosis. *Nature* **513**, 90-94, doi:10.1038/nature13608 (2014).

15 Ma, K. *et al.* Efficient targeting of FATS at a common fragile site in mice through TALEN-mediated double-hit genome modification. *Biotechnol Lett* **36**, 471-479, doi:10.1007/s10529-013-1387-z (2014).

16 Mizuno, S. *et al.* Simple generation of albino C57BL/6J mice with G291T mutation in the tyrosinase gene by the CRISPR/Cas9 system. *Mamm Genome* **25**, 327-334, doi:10.1007/s00335-014-9524-0 (2014).

17 Wang, H. *et al.* One-step generation of mice carrying mutations in multiple genes by CRISPR/Cas-mediated genome engineering. *Cell* **153**, 910-918, doi:10.1016/j.cell.2013.04.025 (2013).

18 Zuckermann, M. *et al.* Somatic CRISPR/Cas9-mediated tumour suppressor disruption enables versatile brain tumour modelling. *Nat Commun* **6**, 7391, doi:10.1038/ncomms8391 (2015).

19 Mandasari, M. *et al.* A facile one-step strategy for the generation of conditional knockout mice to explore the role of Notch1 in oroesophageal tumorigenesis. *Biochem Biophys Res Commun* **469**, 761-767, doi:10.1016/j.bbrc.2015.12.006 (2016).

20 Carbery, I. D. *et al.* Targeted genome modification in mice using zinc-finger nucleases. *Genetics* **186**, 451-459, doi:10.1534/genetics.110.117002 (2010).

21 Khan, M. *et al.* The evolutionarily conserved genes: Tex37, Ccdc73, Prss55 and Nxt2 are dispensable for fertility in mice. *Sci Rep* **8**, 4975, doi:10.1038/s41598-018-23176-x (2018).

22 Liu, Y. M. *et al.* Abnormalities of hair structure and skin histology derived from CRISPR/Cas9-based knockout of phospholipase C-delta 1 in mice. *J Transl Med* **16**, 141, doi:10.1186/s12967-018-1512-9 (2018).
